# Single-Cell RNA Sequencing With Combined Use of Bulk RNA Sequencing to Reveal Cell Heterogeneity and Molecular Changes at Acute Stage of Ischemic Stroke in Mouse Cortex Penumbra Area

**DOI:** 10.3389/fcell.2021.624711

**Published:** 2021-02-22

**Authors:** Kang Guo, Jianing Luo, Dayun Feng, Lin Wu, Xin Wang, Li Xia, Kai Tao, Xun Wu, Wenxing Cui, Yixuan He, Bing Wang, Zhenwei Zhao, Zhiguo Zhang

**Affiliations:** ^1^Department of Neurosurgery, Tangdu Hospital, Fourth Military Medical University, Xi’an, China; ^2^Department of Neurosurgery, West Theater General Hospital, Chengdu, China; ^3^Department of The Central Laboratory, The Second Affiliated Hospital of Xi’an Medical University, Xi’an, China

**Keywords:** single-cell sequencing, general transcriptome, ischemic stroke, brain cortex penumbra, microglia, cell survival

## Abstract

Stroke has been the leading cause of adult morbidity and mortality over the past several years. After an ischemic stroke attack, many dormant or reversibly injured brain cells exist in the penumbra area. However, the pathological processes and unique cell information in the penumbra area of an acute ischemic stroke remain elusive. We applied unbiased single cell sequencing in combination with bulk RNA-seq analysis to investigate the heterogeneity of each cell type in the early stages of ischemic stroke and to detect early possible therapeutic targets to help cell survival. We used these analyses to study the mouse brain penumbra during this phase. Our results reveal the impact of ischemic stroke on specific genes and pathways of different cell types and the alterations of cell differentiation trajectories, suggesting potential pathological mechanisms and therapeutic targets. In addition to classical gene markers, single-cell genomics demonstrates unique information on subclusters of several cell types and metabolism changes in an ischemic stroke. These findings suggest that *Gadd45b* in microglia, *Cyr61* in astrocytes, and *Sgk3* in oligodendrocytes may play a subcluster-specific role in cell death or survival in the early stages of ischemic stroke. Moreover, RNA-scope multiplex *in situ* hybridization and immunofluorescence staining were applied to selected target gene markers to validate and confirm the existence of these cell subtypes and molecular changes during acute stage of ischemic stroke.

## Introduction

Stroke has been the leading cause of adult morbidity and mortality worldwide and in China over the past several years, whether on an ischemic or hemorrhagic base (Rosamond et al., 2008; Wang et al., 2017; Cao et al., 2019). Ischemic stroke accounts for the largest proportion, about 75–80% of all strokes (Xu et al., 2020). Revascularization *via* intravenous thrombolysis and mechanical thrombectomy is a primary therapeutic goal in acute phase ischemic strokes in clinical practice (Mistry et al., 2017). However, the dramatic reduction of cerebral blood flow during the acute stage causes a cascade of events, including energy supply depletion, arrest of metabolic processes, subsequent cell damage, and breakdown of the BBB. Injured and dead cells from damaged area release proinflammatory mediators and cell debris, inducing neuroinflammation, and recruiting peripheral immune cells (Xiong et al., 2016). The rapidly progressive degeneration and dysfunction of neurons and other cells caused by the vascular blockage are critical.

After an ischemic stroke, two distinct areas are present: the infarcted core and the penumbra area (Heiss, 2000). The ischemic penumbra is considered a region with many dormant or reversibly injured brain cells, which may remain viable for several hours due to collateral arteries supplying this region after an ischemic event. The NVU, which maintains the normal physiological functions and repairs damaged cells, includes neurons, astrocytes, microglia, endothelial cells, pericytes, basement membranes, and extracellular matrices. The NVU has been proposed as an entity in stroke and neurodegenerative diseases in past research (Lo and Rosenberg, 2009; Steliga et al., 2020). However, several cell types within the NVU have significantly different responses to ischemic stroke. Furthermore, the causes of heterogeneity of these NVU cells in the stroke penumbra area remain elusive, partly due to technological limitations in studying these cells separately *in vivo*. Previous studies have focused on one cell type *in vivo* or *in vitro* under stroke conditions or in bulk RNA-sequencing. The advent of scRNA-seq has enabled the analysis of cell population heterogeneity at the single-cell level (Cohen et al., 2018; Mickelsen et al., 2019). To date, this study is the first single-cell sequencing research investigating the penumbra pathological process in thousands of physiological and pathological brain cells, offering a cell atlas of the cortex penumbra. In this study, a mouse model of transient focal cerebral ischemia was used. We describe NVU cell heterogeneity, death, and survival under ischemic stroke conditions by analyzing brain tissues from stroke and sham groups at single-cell resolution.

## Materials and Methods

### Mice and Ethics Statement

Twenty-nine healthy male mice (C57BL/6, 10 weeks of age, 20–25 g) were obtained from the Laboratory Animal Center of the Fourth Military Medical University. All experimental procedures were conducted in compliance with the Ethics Committee of the Fourth Military Medical University and the guidelines of the National Institutes of Health Guide for the Care and Use of Laboratory Animals. The mice were kept in a pathogen-free SPF animal room at 18–22°C and 60% humidity under a 12-h light/dark cycle and free access to food and water.

### Animal Model of Middle Cerebral Artery Occlusion

The MCAO model was established as described previously (Lopez and Vemuganti, 2018). First, mice were anesthetized with 2% pentobarbital sodium and fixed on a temperature-regulated heating pad, maintaining the rectal temperature at 37.0 ± 0.5°C during surgery. Laser Doppler flowmetry was used to measure focal cerebral blood flow in all ischemic stroke animals. During the MCAO, if the mouse’s focal cerebral blood flow failed to reach 30% or less than baseline levels or the animal died during postischemic reperfusion, it was excluded from further experiments. As previously described, focal cerebral ischemia was produced for 60 min through intracranial occlusion of the left middle cerebral artery. For the sham-operated group, the same anesthesia and surgical procedures were performed, except for the MCAO. The animals were killed and brains were collected 24 h after ischemic stroke induction. The penumbra area was isolated immediately and made into the cell suspension. The same process was applied to the corresponding brain region of the sham-operated group.

Of the 29 mice used in this experiment, 12 sham-operated mice and 12 ischemic mice were included in further tests; four mice were excluded due to failure to induce ischemia or peri-ischemic death. Brains from six sham-operated and six ischemic mice were collected for scRNA-seq processing; six sham-operated and six ischemic mice were used for bulk RNA-seq processing; one sham operated and one ischemic mouse were used for RNAscope multiplex *in situ* hybridization and immunofluorescence staining.

### Brain Tissue Dissociation

All mice were rapidly decapitated for brain tissue under anesthesia of 2% pentobarbital sodium. The brain tissue was cut into approximately eight coronal slices or 0.5 cm sections using a sterile scalpel. Preparation of a single-cell suspension of brain tissue was performed with an adult mouse brain dissociation kit (MACS, Miltenyi Biotec Inc., Auburn, CA, United States) according to the manufacturer’s instructions. Briefly, the isolated penumbra was finely cut into pieces and dissociated in enzyme mix solution (50 μl enzyme P, 1,900 μl buffer Z, 10 μl enzyme A, and 20 μl buffer Y) for 30 min at 37°C. A strainer (70 μm) was placed on a 50-ml tube and used to filter the tissue fragments. The cell suspension was centrifuged at 300 × *g* for 10 min at 4°C, and the supernatant was aspirated completely. Debris removal reagent [3,100 μl cold D-PBS, 900 μl cold debris removal solution, and 4 ml overlay D-PBS] was added to resuspend the cell sample. The suspension was then centrifuged again at 4°C and 3,000 × *g* for 10 min with full acceleration and full brake to remove cell debris. The cells were resuspended in 1 ml cold 1 × red blood cell removal solution and incubated for 10 min at 2–8°C. The solution was centrifuged at 4°C and 300 × *g* for 10 min to remove red blood cells. The resuspended cells were processed immediately for scRNA-sequencing.

### scRNA-Sequencing Processing

Cell viability was evaluated by trypan blue staining, and the samples with cell viability >90% were processed with Single Cell 3’ v3 Reagent Kit (v3 Chemistry) (10× Genomics, Pleasanton, CA, United States), according to the manufacturer’s instructions. The single cell in the prepared cell suspensions were then encapsulated in droplets using 10× Genomics GemCode Technology. In the droplets, the mRNA was released by cell lysis and attached to the UMI and other primer sequences of the gel beads, forming single-cell GEM structures (gel bead in emulsions). The mRNA underwent a reverse transcription reaction in a droplet to form cDNA, with subsequent cDNA library construction. The Chromium i7 Multiplex Kit (10× Genomics; PN-120262) was used for library construction. Quality control was performed using the Qubit 4 Fluorometer (Thermo Fisher; Q33227) and the Agilent 2100 Bioanalyzer. The resulting prepared cDNA library was sequenced on a NovaSeq 6000 2 × 150 bp, and ∼50,000 read pairs per cell were obtained.

**FIGURE 1:**
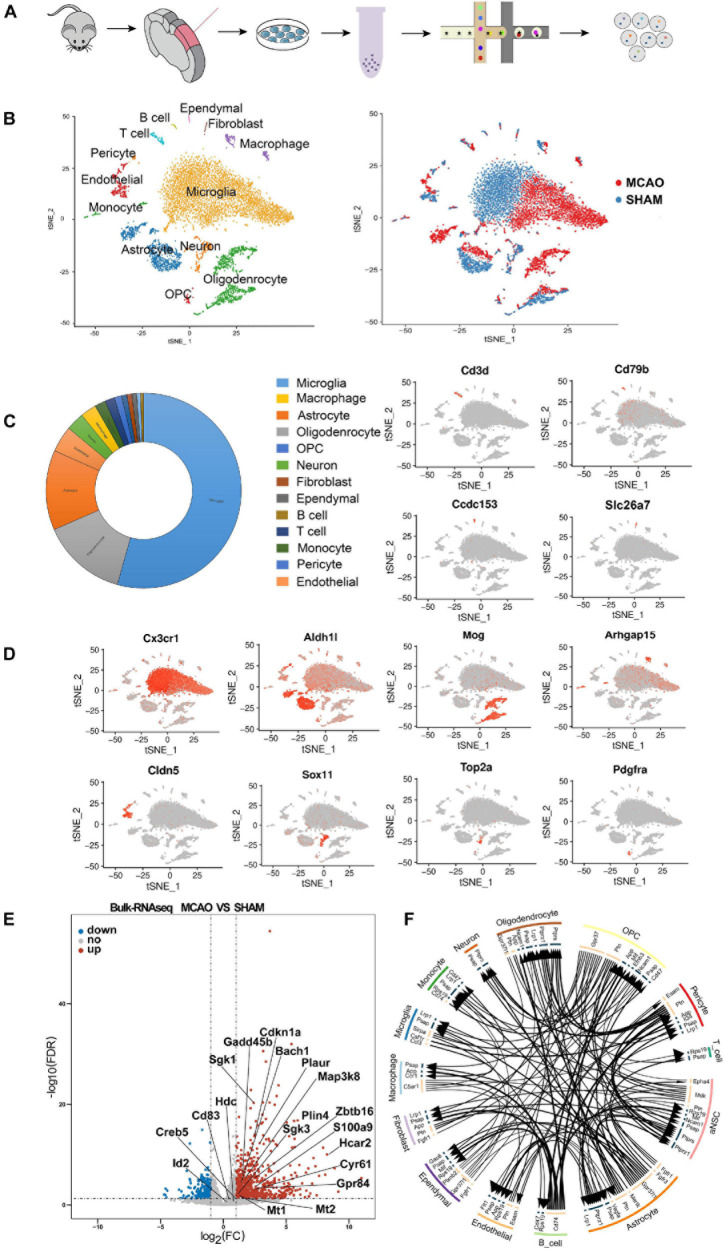
Overview of the 18,273 single cells from MCAO and sham groups. **(A)** Schematic flow of single-cell sequencing. **(B)** t-SNE plot of the 18,273 cells with the associated cell type (left) and its sample type of origin (right). **(C)** Toroidal diagram of composition ratio with 13 cell types. **(D)** Expression of marker genes for the partial cell types previously identified. The additional marker genes for each cell type are shown in Supplementary File 1. **(E)** Volcano plot showing the differential genes obtained with the scRNA-seq and validated by the bulk RNA-seq results. **(F)** Intercellular receptor ligands between cell types are presented in a circle diagram.

The scRNA-seq data were processed through the 10× Genomics Cell Ranger Single Cell 3.0.1 pipeline using default and recommended parameters. The Seurat (v1.4.0.15) R toolkit was used for quality control of the following scRNA-seq experiment in this study. For each sample, the data with low-quality reads (<500 or >4,000 total expressed genes) or unrelated sequences (ribosomal genes and >15% mitochondria-expressed genes) were excluded from downstream analysis. The schematic flow of single-cell sequencing is shown in Figure 1A.

Since the cell number of a single sample from the penumbra was insufficient for sequencing, we mixed six samples from MCAO mice and six samples from sham mice into two cell suspension pools. After batch effects removal, low-quality data filtration, and data normalization, a total of 18,273 cells (8,623 MCAO, 9,650 sham) were included in the downstream analysis (Supplementary Figures 1A–C). Then, PCA and t-SNE were performed for dimensionality reduction, and cell clusters were defined. PCA and t-SNE were performed (PCs 1 through 24) using the R package Seurat (version 2.3.1). The FindAllMarkers function in Seurat and Wilcoxon rank-sum test were used to identify the marker genes of each transcriptional cluster, which were determined with a minimum log-fold change threshold of 0.25 and *P*-values < 0.05. In addition, CellTalker was used to elucidate the intercellular receptor-ligand changes between cell types (Figure 1F).

### Bulk RNA-Sequencing Processing

The penumbra of brains from six sham-operated and six ischemic mice were collected for bulk RNA-seq processing. Briefly, total RNA was extracted using Trizol reagent kit (Invitrogen, Carlsbad, CA, United States) according to the manufacturer’s protocol. RNA quality was assessed on an Agilent 2100 Bioanalyzer (Agilent Technologies, Palo Alto, CA, United States) and examined using RNase free agarose gel electrophoresis. After extraction of total RNA, eukaryotic mRNA was enriched by Oligo(dT) beads, and prokaryotic mRNA was enriched by removing rRNA with Ribo-Zero TM Magnetic Kit (Epicentre, Madison, WI, United States). The enriched mRNA was then fragmented into short fragments using fragmentation buffer and reverse transcribed into cDNA using random primers. Second-strand cDNA were synthesized by DNA polymerase I, RNase H, dNTP, and buffer. Then the cDNA fragments were purified with QiaQuick PCR extraction kit (Qiagen, Venlo, Netherlands), end repaired, A base added, and ligated to Illumina sequencing adapters. The size of the ligated product was selected by agarose gel electrophoresis, PCR amplified, and sequenced using Illumina Novaseq6000. To get high-quality clean reads, reads were further filtered by fastp (version 0.18.0). RNAs differential expression analysis was performed by DESeq2 software between two different groups (and by edgeR between two samples). The genes/transcripts with the parameter of FDR below 0.05 and absolute fold change ≥ 2 was considered differentially expressed genes/transcripts.

### RNAscope *in situ* Hybridization

RNAscope multiplex *in situ* hybridization [Advanced Cell Diagnostics (ACD), Newark, CA, United States] was applied to evaluate and localize the target genes in the microglia cells. Each experimental step was performed according to manufacturer’s instructions. Fresh mice brain frozen sections (15 μm) placed on glass slides (Fisher Scientific, Waltham, MA, United States) were immersed in a 4% paraformaldehyde solution containing 1% diethyl pyrocarbonate (DEPC) for 15 min. After three rinses with sterile PBS, gradient dehydration of 50, 75, and 100% ethanol was performed for 5 min per step. Then, dehydration was repeated in 100% anhydrous ethanol, and the sections dried at room temperature (RT) for 5 min. Afterward, the slides were incubated with RNAscope hydrogen peroxide for 10 min at RT. After rinsing three times with sterile PBS, the slides were dried at room temperature for 5 min. A slide holder was filled with 200 ml of RNAscope 1 × Target Retrieval Reagent and incubated with the slides at 100°C for approximately 15 min; the slides were then washed immediately with sterile PBS. An appropriately sized hydrophobic circle was drawn around the brain slice on the slides with an Immedge hydrophobic barrier pen (Vector Laboratories, Burlingame, CA, United States). The dry slides were loaded into the RNAscope EZ-Batch Slide Holder, adding three to five drops of Protease IV to cover the sections completely. The sections were incubated with Protease IV for 30 min at 40°C, then rinsed in sterile PBS with slight agitation for 2 min, adding three to four drops of the appropriate probe mix to cover each section entirely. The tray was closed and inserted into the HybEZ oven (ACD) for 2 h at 40°C. The four targeting probes were the *Gadd45b* (GenBank accession number NM_008655.1, LOT:20198A, ACD), *Hcar2* (GenBank accession number NM_030701.3, LOT:20198B, ACD), and *Gpr84* (GenBank accession number NM_030720.2, LOT:19354A, ACD) in channel C1, and *C1qb* (GenBank accession number NM_009777.3, LOT:20198A, ACD) in channel C2. The slides were washed for 2 min three times with wash buffer at RT, then incubated with four to six drops of RNAscope Multiplex AMP 1 (40°C for 30 min), AMP 2 (40°C for 30 min), and AMP3 (40°C for 15 min). We used a nucleic acid fluorescent dye (Opal 520, CAT. NO. ASOP520, Asbio) to mark channel C1 as green and nucleic acid fluorescent dye (CAT. NO. ASOP570, Asbio) to mark channel C2 as red. The slides were washed in 1× washing buffer twice between incubations, then incubated with three to five drops of DAPI for 30 s at RT. Fluorescent images were captured blindly using an A1 Si confocal microscope (Nikon, Tokoyo, Japan).

### Immunofluorescence Staining

Immunofluorescence staining was applied to localize the target genes in astrocytes, oligodendrocytes, and macrophages. The mouse brains were perfused with PBS and then fixed with 4% paraformaldehyde for 24 h, dehydrated at 20 and 30% of ethanol, and frozen for slicing into 30 μm sections. After incubation with PBS solution containing 0.1% Triton X-100 for 30 min and blocked with PBS containing 5% goat serum (Gibco, Gaithersburg, MD, United States) for 30 min, fresh brain sections were incubated with primary antibodies overnight at 4°C, then with secondary antibodies (1:250) for 2 h, and finally with DAPI 1:1,000 (Sigma-Aldrich, St. Louis, MO, United States) for 15 min at room temperature. The primary antibodies used were as follows: goat anti-chicken glial fibrillary acidic protein (*GFAP*) (1:200; Mouse, Invitrogen, Carlsbad, CA, United States), *Cyr61* (1:500; Rabbit, Novusbio, Littleton, CO, United States), *Oligo2* (1:500; Mouse, Proteintech, Rosemont, IL, United States), *Sgk3* (1:100; Rabbit, Proteintech), *Arhgap15* (1:200; Rabbit, Proteintech), and *S100a9* (1:200; goat, R&D Systems, Minneapolis, MN, United States). The secondary antibodies used were Alexa Fluor 594 donkey anti-chicken IgG (Invitrogen), donkey anti-rabbit IgG (Alexa Fluor 488), donkey anti-mouse IgG (Alexa Fluor 594), donkey anti-rabbit IgG (Alexa Fluor 594), and donkey anti-goat IgG (Alexa Fluor 488). All images were captured blindly using an A1 Si confocal microscope (Nikon).

### Statistical Analysis

For the differentially expressed genes, DESeq and DESeq2 packages were used to determine the underlying distributions for the read counts. We used Benjamini–Hochberg correction for FDR and Bonferroni correction for *P*-value based tests. The statistical differences between the two groups in bulk RNA-seq were examined using Student’s *t*-test (unpaired, two-tailed). *P* < 0.05 were considered statistically significant. The statistical analysis was performed with GraphPad Prism 8.0 software (GraphPad, Inc., San Diego, CA, United States).

## Results

### Unbiased Identification of Cell Identities of Brain Cortex Penumbra Region With scRNA-Seq

We identified 24-cell clusters, each containing similar gene expression patterns (Supplementary File 1). We compared the cluster-specific gene signatures obtained with known signatures of mouse cortex cell types reported in previous studies (Moffitt et al., 2018; Saunders et al., 2018; Zeisel et al., 2018) and the CellMarker database^1^. All of these cells were classified into 13 main cell lineages (Figure 1B). We identified the major cell types, including neurons, astrocytes, microglia, oligodendroglia, OPC, endothelial cells, pericytes, macrophages, B cells, T cells, monocyte cells, ependymal cells, and fibroblasts (Figure 1D and Supplementary File 1). We also discovered some novel gene signatures, which may be valuable MCAO cell-specific markers for future analysis (Supplementary File 1). Notably, among the 13 cell types identified, the majority of cells were microglia, astrocytes, and oligodendrocytes (Figure 1C).

When comparing MCAO and sham samples, we note that many microglia, astrocytes, macrophages, oligodendrocytes, and endothelial cell subclusters were more enriched in the MCAO sample (Figure 1B), which indicates that these cells from the MCAO group underwent some significant alterations. We performed further clustering on these cell types (i.e., microglia, astrocytes, macrophages, neurons, endothelial cells, and oligodendrocytes) to evaluate subcluster heterogeneity. At the end of the experiment, we confirmed the subcluster-specific expression patterns in the penumbra. We also validated the expression specificity of several novel markers with RNAscope multiplex *in situ* hybridization and immunofluorescence staining.

Since only a few cells had cell proliferation markers, we did not correct the cell cycle and subject them to the next step of data analysis (Lambrechts et al., 2018). Indeed, the enzymatic method used in the lysis of brain tissue to obtain single-cell suspensions may result in the loss of neuronal cells, potentially causing underrepresentation of neurons in the total cell population obtained and changes in enzymatic-related genes. A previous study suggested that the cell isolation procedure may present cell dissociation artifacts (van den Brink et al., 2017). Therefore, to exclude the effect of enzymatic digestion and reduce method-specific errors in single-cell sequencing, we added bulk RNA-seq as a supplementary method to corroborate the findings (Figure 1E). TPM values were used as a criterion to determine gene expression in bulk RNA-seq (detailed information is reported in Supplementary File 8). These combined data demonstrate high intercellular heterogeneity in cortex penumbra cells during MCAO and suggest that these 13 cell subtypes include most cellular heterogeneity in the mouse cortex brain.

### Microglia Cells Are Prevalent in Acute MCAO Stage and Exhibit Polarization

**FIGURE 2:**
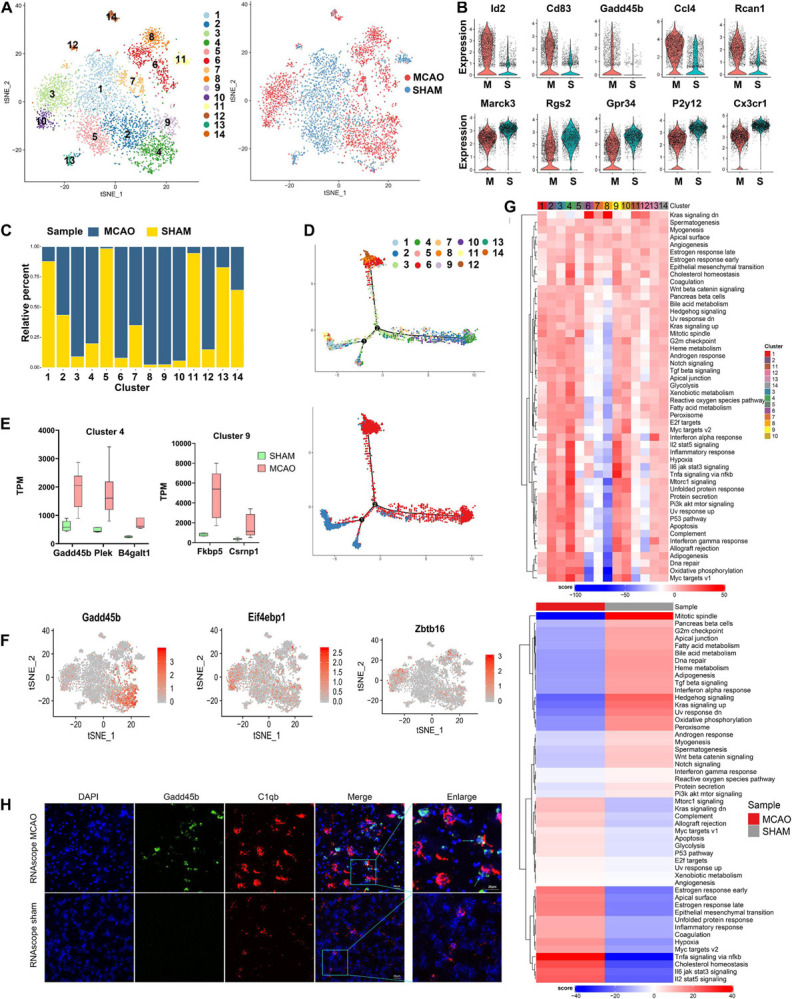
Microglia cells in MCAO and sham groups. **(A)** t-SNE plot of 4440 microglia cells with the associated cell subcluster (left) and its sample type of origin (right). **(B)** Violin plot showing the top 10 differential microglia expression genes between MCAO and sham groups. **(C)** Proportion of each cell subcluster in the MCAO and sham groups. **(D)** Differentiation trajectories of microglia subclusters in ischemic stroke. **(E)** Validation of scRNA-seq results for differential expression genes in subclusters 4 and 9 of microglial cells in bulk RNA-seq. **(F)** t-SNE plot of some genes (such as Gadd45b, Eif4ebp1, and Zbtb16) exhibits cell subcluster specificity in the MCAO group. **(G)** Differences in pathway activities between MCAO and normal microglia cells and between subclusters scored per cell by GSVA. **(H)** The RNAscope images for MCAO and sham groups display cell colocalization of Gadd45b (green) and the corresponding cell marker C1qb (red).

To further investigate microglia diversity at early stage of ischemic stroke, 5,108 microglia cells from MCAO and sham samples were further reclustered into 14 subclusters for downstream analysis (Figure 2A). Differential gene expression analysis identified numerous genes specifically enriched in each subcluster (Supplementary Figure 2B). These subclusters revealed significant variability between the MCAO and sham groups. Based on the cell subpopulation proportional diagram (Figure 2C), subclusters 3, 4, 6, 8, 9, and 10 were mainly enriched in MCAO samples, while subclusters 1, 5, 11, and 13 were more enriched in sham samples. In particular, we noticed that the genes *Id2*, *Cd83*, *Gadd45b*, *Ccl4*, and *Rcan1* were overexpressed in the MCAO group, while *Marcks*, *Rgs2*, *Gpr34*, *P2ry12*, and *Cx3cr1* were downregulated, compared with the sham samples (Figure 2B).

In addition to comparing differentially expressed genes (DEGs), we employed GSVA to observe the response of each microglia subcluster under hypoxic conditions. Interestingly, subclusters 3, 4, 6, 8, 9, and 10 exhibited different functional variations in their early response to stroke, although these subclusters were all more enriched in MCAO samples (Figure 2G). Subclusters 3, 4, 9, and 10 showed more similar functional changes, while subclusters 6 and 8 seemed to be quite different during the early stage of stroke response. Furthermore, a similar trend was also identified in the pseudo-time trajectory diagram (Figure 2D). It seemed that subclusters 3, 4, 9, and 10 and subclusters 6 and 8 followed different differentiation trajectories, indicating that these two groups of subclusters might be M1- and M2-type microglia activation states. We discovered that some genes (such as *Gadd45b* in subclusters 4 and 9, *Eif4ebp1* in subcluster 10, and *Zbtb16* in subcluster 3) were subcluster specific, and these genes were specifically enriched in the MCAO group (Figure 2F). Additionally, we applied the differential genes of subclusters 4 and 9 (*Gadd45b*, *Plek*, and *B4galt1* in subcluster 4, and *Fkbp5* and *Csrnp1* in subcluster 9) into bulk RNA-seq for further validation (Figure 2E).

Meanwhile, the GSVA demonstrated that the most enriched signatures in subclusters 3, 4, 9, and 10 were hypoxia, TNF-α-, IL- 6-, and IL-2-enriched inflammation-related genes and pathways, suggesting that these subclusters experienced an intense inflammatory response. However, subclusters 6 and 8 were mainly enriched in the Kras signaling pathway, closely associated with the survival of cancer cells in previous studies (Saad et al., 2019). These microglia subclusters may have higher survival chances than other subclusters under an ischemic stroke owing to this pathway. In addition, the characteristic marker genes of M2-type microglia (such as *Arg1*, *Ym1*) had not been fully expressed yet, indicating that the differentiation of M2-type microglia is delayed compared with M1 type at the early stage of a stroke. The microglia results also revealed that other genes (such as *Iba1* and *P2y12*) are hypoexpressed in subclusters 6 and 8. A previous study suggested that the *P2y12* knockout may help to reduce neuronal death and microglia accumulation in penumbra (Webster et al., 2013).

Next, we performed GO and KEGG analysis of these DEGs. The results indicated that these DEGs were most enriched in transcriptional mis-regulation (*Gadd45b*, *Id2*, *Cebpb*), toll-like receptor signaling pathway (*Ccl4*), and IL-17 signaling pathway (*Cebpb*, *NF-*κ*Bia*) (Supplementary Figures 2C–E). Besides, the metabolic changes identified in these subclusters are most functional in phagocytic vesicles and inflammatory responses. Furthermore, RNAscope *in situ* hybridization validated the existence and location of subcluster-specific DEGs (Figure 2H and Supplementary Figure 2A).

### Peripheral Macrophages Are Fully Activated During the Acute Phase

**FIGURE 3:**
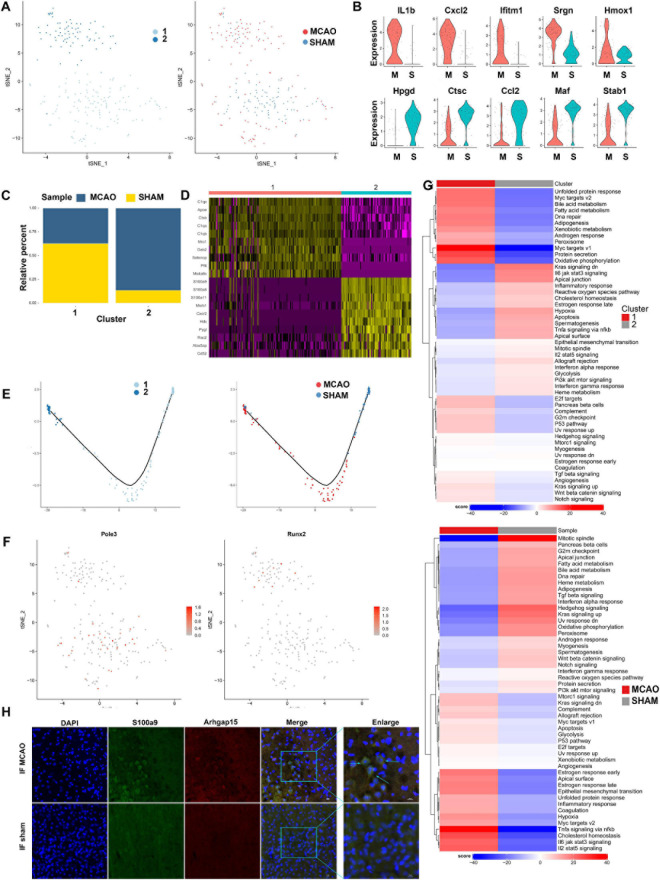
Peripheral macrophage cells in MCAO and sham groups. **(A)** t-SNE plot of 217 peripheral macrophages with the associated cell subcluster (left) and its sample type of origin (right). **(B)** Top 10 differential expression genes of peripheral macrophages in MCAO and sham groups presented in a violin plot. **(C)** Proportion of each cell subclusters in MCAO and sham groups. **(D)** Heatmap of top 10 differential expression genes in each cell subpopulation. **(E)** Distinctive differentiation trajectories of macrophage subclusters in ischemic stroke. **(F)** t-SNE plots of SCENIC analysis of each cell subcluster showing that transcription factor Pole3 and Runx2 are mainly enriched in subclusters 1 and 2, respectively. **(G)** Differences in pathway activities scored per cell by GSVA between MCAO and normal peripheral macrophages and between subclusters. **(H)** The immunofluorescence images for MCAO and sham groups display cell colocalization of S100a9 (red) and the corresponding cell marker Arhgap15 (green).

We detected 217 peripheral macrophages after stroke induction. Twenty-four hours after the acute stage of an ischemic stroke, peripheral macrophages were recruited around the penumbra and polarized more completely into two subclusters, compared with microglia (Figure 3A). In contrast to the sham group, we noticed that *IL-1b*, *Cxcl2*, *Ifitm1*, *Srgn*, and *Hmox1* genes were upregulated in the MCAO group, while *Hpgd*, *Ctsc*, *Ccl2*, *Maf*, and *Stab1* genes were downregulated (Figure 3B). Moreover, the subclusters of peripheral macrophages exhibited sample specificity, with subcluster 2 being largely prevalent in the MCAO group (Figure 3C). Differential gene expression analysis identified numerous genes specifically enriched in these subclusters (Figure 3D). The pseudo-time trajectory diagram shows that the two subclusters developed following contrasting trajectories (Figure 3E). The GSVA showed that subcluster 1 was mainly enriched in Myc targets v1, protein secretion, and oxidative phosphorylation. In contrast, subcluster 2 was mostly enriched in TNF-α signaling *via* NF-κB, hypoxia, and IL-6/JAK/STAT3 signaling pathway, suggesting that this subcluster is proinflammatory (Figure 3G). We also identified several proinflammatory-related genes (*S100a9*, *S100a8*, *Mmp9*, and *Mmp8*) highly expressed in this subcluster; this finding was validated by bulk RNA-seq analysis (Supplementary Figure 3C). These genes are associated in particular with inflammation and the BBB. Previous studies suggest that physical BBB disruption can be caused by matrix metalloprotease through the digestion of BBB matrix proteins. Therefore, peripheral macrophages, as well as microglia and astrocytes, may be responsible for disrupting the BBB.

By analyzing the GO and KEGG of macrophage genes that were different between the MCAO and sham groups, we revealed that the main pathways activated during a stroke are functional immune processes, such as response to wounding, cell migration, defense response, cell motility, inflammatory response, exosomes, phagosome, Hif-1 signaling pathway, and TNF signaling pathway (Supplementary Figure 3B). Moreover, another enrichment result revealed that subcluster 2 was mainly oriented to metabolic functions such as glycolysis and gluconeogenesis, histidine, and retinol metabolism, urea cycle, and glycogen degradation, showing metabolic changes under ischemic stroke conditions. Detailed metabolic alterations are shown in Supplementary Figure 3E.

Single-cell regulatory network inference and clustering (SCENIC) analysis (Aibar et al., 2017) revealed that transcription factors, such as *Runx2*, were highly expressed in the MCAO group, while *Pole3* was upregulated in the sham group (Figure 3F). These transcription factors exhibited significant variance in peripheral macrophage subclusters during the early stage of ischemic stroke. Finally, we validated the *S100a9* gene expression through immunofluorescence staining of frozen sections of mouse brain tissue. We found that peripheral macrophages were recruited more abundantly around the penumbra zone and infarct area (Figure 3H and Supplementary Figure 3A).

### Astrocyte Heterogeneity

**FIGURE 4:**
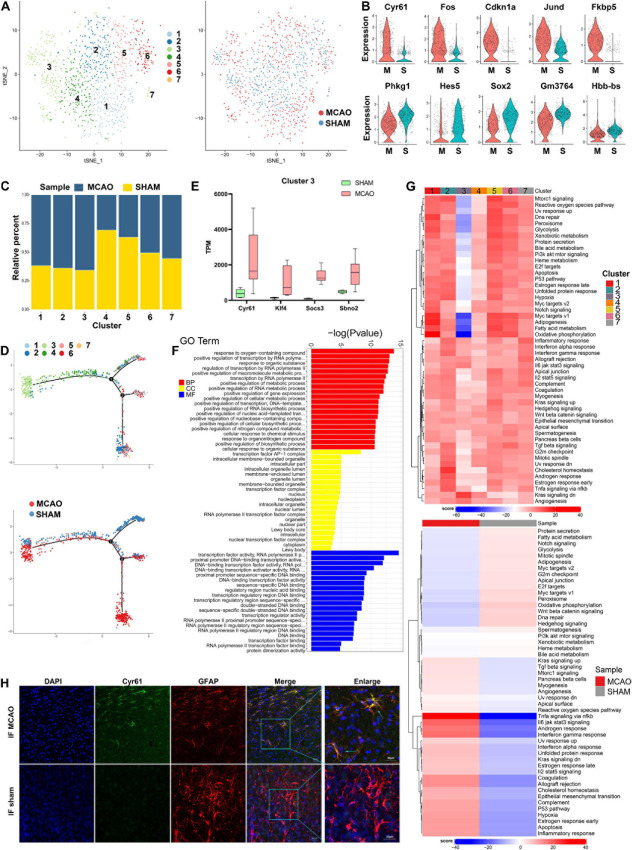
Astrocyte cells in MCAO and sham groups. **(A)** t-SNE map of 1,083 astrocytes with the associated cell subcluster (left) and its sample type of origin (right). **(B)** Violin plot showing the top 10 differential astrocyte expression genes between MCAO and sham groups. **(C)** Proportion of each cell subclusters in MCAO and sham groups. **(D)** Distinctive subclusters differentiation trajectories in ischemic stroke. **(E)** Validation of scRNA-seq results for differential expression genes in astrocyte subcluster 3 in bulk RNA-seq. **(F)** GO enrichment analysis of astrocyte differential expression genes. **(G)** Differences in pathway activities scored per cell by GSVA between MCAO and normal astrocytes and between subclusters. **(H)** The immunofluorescence images for sham and MCAO groups display cell colocalization of Cyr61 (green) and the corresponding cell marker GFAP (red).

Within the 1,083 astrocytes detected after stroke induction, seven subclusters were identified. The t-SNE plots of astrocyte subclusters in MCAO and sham groups are shown in Figure 4A. The number of cells in subcluster 7 was less than 30; therefore, this subcluster was excluded from downstream analyses. Compared with the sham group, genes such as *Cyr61*, *Fos*, *Cdkn1a*, *Jund*, and *Fkbp5* were upregulated in the MCAO group, while *Phkg1*, *Hes5*, *Sox2*, *Gm3764*, and *Hbb-bs* were downregulated (Figure 4B). As seen from the ratio diagram (Figure 4C), the changes in the proportion of astrocyte subcluster composition between MCAO and sham groups were not as significant as in microglia and macrophages. Nonetheless, subcluster 3 exhibited greater diversity than the other subpopulations in GSVA and the pseudo-time trajectory (Figures 4D,G). This subcluster is mostly enriched in the Kras signaling pathway, producing a positive effect during the subsequent stroke stage. The functional metabolic enrichment in subcluster 3 also varies significantly from other subclusters (Supplementary Figure 4F), with *O*-glycan biosynthesis and thiamine metabolism mostly enriched. Furthermore, we revealed some genes (*Cyr61*, *Klf4*, *Socs3*, and *Sbno2*) that are specifically expressed in this subcluster and validated these genes in bulk RNA-seq analysis (Figure 4E). However, the role of the *Cyr61* gene in ischemic stroke is still unclear.

The results of GO and KEGG enrichment analysis indicated that astrocyte responses in early stroke are dominant in the intracellular region, especially in the positive regulation of metabolic processes and gene expression, transcription factor AP-1 complex, and transcription factor activity (Figure 4F). The most involved pathways are MAPK signaling pathway, estrogen signaling pathway, and toll-like receptor signaling pathway (Supplementary Figures 4C,D). In the SCENIC analysis, *Bach1* and *Zcchc14* were highly expressed in the MCAO group, whereas *Ep300*, *Mxi1*, and *Rbbp5* were overexpressed in the sham group (Supplementary Figure 4G). Immunofluorescence staining of frozen sections of mouse brain tissue validated the *Cyr61* expression differences between MCAO and sham groups (Figure 4H and Supplementary Figure 4A).

### Oligodendrocytes Are Mainly Enriched in MCAO Group and Exhibit Heterogeneity

**FIGURE 5:**
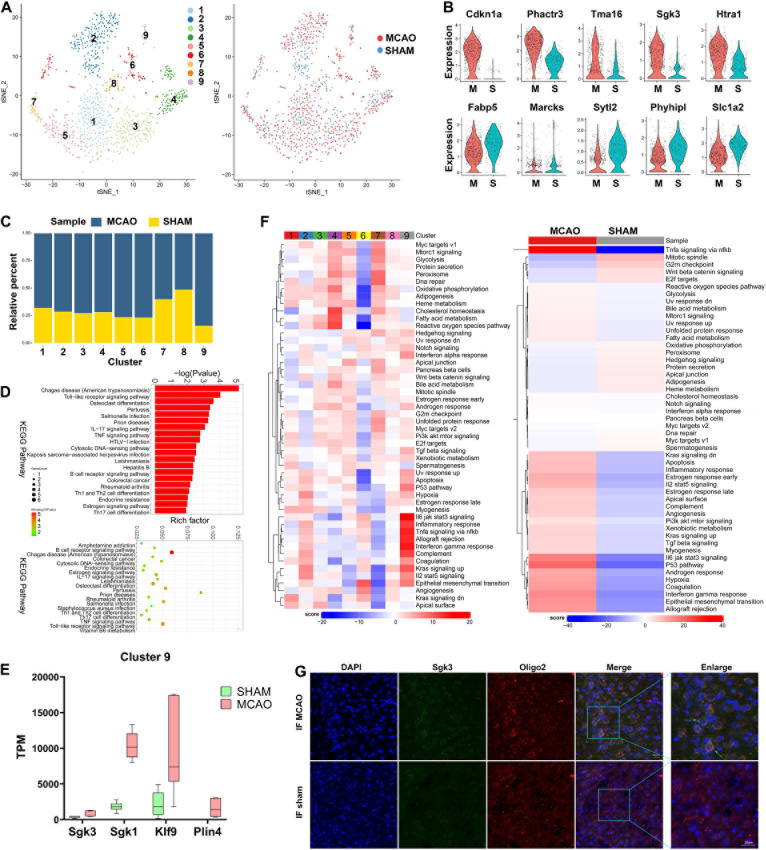
Oligodendrocyte cells in MCAO and sham groups. **(A)** t-SNE map of 1154 oligodendrocytes with the associated cell subcluster (left) and its sample type of origin (right). **(B)** Violin plot showing the top 10 differential oligodendrocyte expression genes between MCAO and sham groups. **(C)** Proportion of each cell subclusters in MCAO and sham groups. **(D)** GO and KEGG enrichment analysis of oligodendrocyte differential expression genes. **(E)** Validation of scRNA-seq results for differential expression genes in subcluster 9 in bulk RNA-seq. **(F)** Differences in pathway activities scored per cell by GSVA between MCAO and normal oligodendrocytes and between subclusters. **(G)** The immunofluorescence images for sham and MCAO group display cell colocalization of Sgk3 (green) and the corresponding cell marker Oligo2 (red).

To date, oligodendrocytes in ischemic stroke have not been studied as intensively as microglia and astrocytes. The role of oligodendrocytes in stroke and the extent of their heterogeneity remain elusive. Among the 1,154 oligodendrocyte cells identified after stroke induction, 9 subclusters were identified (Figure 5A). Compared with the sham group, *Cdkn1a*, *Phactr3*, *Tma16*, *Sgk3*, and *Htra1* were found to be overexpressed in the MCAO group, while *Fabp5*, *Marcks*, *Sytl2*, *Phyhipl*, and *Slc1a2* were underexpressed (Figure 5B). The cell subcluster proportional diagram demonstrates that almost all subclusters were more enriched in the MCAO group, especially subcluster 9 (Figure 5C). Through gene expression analysis, we identified numerous differentially expressed genes specifically enriched in each subcluster (Supplementary File 5). We found that some novel differentially expressed genes (such as *Sgk3*, *Sgk1*, *Klf9*, and *Plin4* in subcluster 9 and *Klk6*, *Arrdc2*, and *Anln* in subcluster 4) were significantly overexpressed. In addition, we validated the expression of these genes with bulk RNA-seq (Figure 5E and Supplementary Figure 5D). Next, we performed GO and KEGG enrichment analyses on these DEGs. The results suggest that oligodendrocyte function is predominantly enriched in response to oxygen-containing compounds, neurotransmitter transporter activity, and L-glutamate and acidic amino acid transmembrane transporter activity (Figure 5D and Supplementary Figures 5B,G).

Based on GSVA, the intersubcluster differences vary significantly; subcluster 9 is especially enriched in inflammatory-related pathways (Figure 5F). Inflammatory response, interferon-gamma response, complement, and IL-6/JAK/STAT3, TNF-α-NF-κB, and IL-2 stat5 signaling were the most enriched pathways in subcluster 9, as well as in the MCAO group overall. In addition, the metabolic diagram of each subcluster showed significant variance; subcluster 2 was mainly enriched in carbonic acid and creatine metabolism, while subcluster 4 in bysteroid metabolism (Supplementary Figure 5E). The above data suggest that these metabolites undergo significant changes in oligodendrocytes during the early stage of ischemic stroke. The trajectory differentiation diagram of oligodendrocyte subclusters does not show significant polarization (Supplementary Figure 5C). SCENIC analysis showed that transcription factors, such as *Nr2f1*, *Arid3a*, and *Gtf3a*, are highly expressed in the MCAO group (Supplementary Figure 5F). However, these transcription factors are subpopulation specific. Immunofluorescence staining confirmed the *Sgk3* gene expression seen in scRNA-seq and bulk RNA-seq results (Figure 5G and Supplementary Figure 5A).

### Neurons and Endothelial Cells Heterogeneity

The 354 endothelial cells detected were reclustered into five different subclusters (Supplementary Figure 6A). In endothelial cells, the upregulated genes (*Tmem252*, *Akap12*, *Mt1*, *Mt2*, and *Lcn2*) and downregulated genes (*Hmcn1*, *Tfrc*, *Car4*, *Slc16a1*, and *Tgfb2*) in the MCAO group are shown in the violin plot and volcano plot (Supplementary Figure 6B). No distinctive changes were observed in the proportions of each subcluster between the MCAO and sham groups (Supplementary Figure 6C). Nevertheless, the GSVA showed a significant difference between subcluster 3 and other subclusters, indicating that subcluster 3 is more functional in epithelial-mesenchymal transition and myogenesis (Supplementary Figure 6G).

Functional enrichment of GO and KEGG from subcluster 3 also exhibited variance, mainly oriented to vasculature development, exosomes, and extracellular matrix (Supplementary Figure 6E). These results suggest that this subcluster is involved in vascular reconstruction and extracellular communication after injury. The processes mainly activated in subcluster 3 were oxidative phosphorylation, ROS detoxification, glutathione metabolism, and cyclic nucleotide metabolism. Moreover, compared with sham samples, the main metabolic changes in acute ischemic stroke in endothelial cells were in transport, Golgi apparatus, glycolysis and gluconeogenesis, porphyrin, and heme metabolism. Ferroptosis, mineral absorption, apoptosis, and HIF-1 signaling pathway were also mainly activated in the MCAO group.

A total of 246 neurons and six subclusters, derived from MCAO or sham tissues, were detected (Supplementary Figure 6H). In contrast to the sham group, we found that genes such as *Mt1*, *Mt2*, *Gfap*, *Ccl4*, and *Ay036118* were highly expressed in the MCAO group, while *Gm17750*, *Nr2f1*, *Stmn2*, *Basp1*, and *Cd24a* were reduced (Supplementary Figure 6I). GABAergic neurons (gamma-aminobutyric acid) were mainly distributed in subclusters 1, 2, and dopamine (DA) neurons in subcluster 5. Subclusters 5 and 6 were discarded from the analysis due to an insufficient number of cells (<30). Detailed marker genes of each neuron subcluster are reported in Supplementary File 7. The proportion of neuron subclusters states that subcluster 4 was mainly enriched in MCAO samples (Supplementary Figure 6J). The metabolism of this cluster was enriched primarily on fatty acid metabolism, glycolysis and gluconeogenesis, and glycine, serine, threonine, alanine, aspartate, and histidine metabolism (Supplementary Figure 6O). From GO and KEGG analysis, we also found that in the MCAO group, neuronal changes were oriented to the nervous system development, neurogenesis, developmental process, cell differentiation, exosomes, extracellular vesicles, axon, and neuron particles (Supplementary Figures 6K,L).

Other cell types (such as B cells, T cells, monocytes, ependymal cells, pericytes, OPCs, and fibroblasts) were isolated in insufficient numbers, and further reclustering would have led to unreliable sequencing results (Lambrechts et al., 2018); therefore, further analysis was not performed.

## Discussion

Intensive research has been conducted on different cell types, such as astrocytes, microglia, macrophages, and oligodendrocytes at the early stages of ischemic stroke (Liu and Chopp, 2016; Jian et al., 2019; Gharbi et al., 2020; Jackson et al., 2020). However, these studies investigated only a single cell type or are based on whole tissue samples (the bulk-RNA sequencing). In addition, intercellular contact in the early stages of stroke and cellular heterogeneity in the penumbra remain elusive. In the past, technological limitations have restricted studies to target a particular cell type, whereas bulk-RNA studies may have missed the different phenotypic changes of a particular cell type in ischemic stroke. We applied scRNA-seq in combination with bulk RNA-seq analysis to investigate the heterogeneity of each cell type in stroke early stages and to detect early therapeutic targets that may help cell survival, studying the mouse brain penumbra. To better understand the early stages of ischemic stroke, we reclustered specific cell types to investigate heterogeneity within different cell types as well as heterogeneity within different subclusters of selected cell type. Furthermore, RNAscope multiplex *in situ* hybridization and immunofluorescence were applied to confirm the existence of these cell subclusters and molecular changes in acute stroke.

Glial cells are always the first line of response when brain blood flow is interrupted (Xu et al., 2020); in line with this finding, we noticed that microglia account for the largest proportion of the number of cells after MCAO induction. However, whether microglia cell polarization promotes inflammation or injury repair varies depending on the pathology conditions (Kitamura et al., 2004). In addition, there is still debate regarding the moment of initial microglia polarization after a stroke (Al-Ahmady et al., 2019). Previous studies have found no significant microglial polarization within 24 h of ischemia induction (Girard et al., 2013; Kanazawa et al., 2017). In contrast to these studies, our single-cell sequencing results demonstrated that microglia already shows polarization and differentiation in two different progression trajectories 24 h after MCAO. These findings mean that the first 24 h may be a good window for an intervention to help cell survival. In the clinical setting, the treatment of venous thrombolysis revascularization is generally considered to have a limited optimal time window (Shen et al., 2020). Interventions to revascularize blood vessels within this time window may save brain tissue in the ischemic penumbra (Yang et al., 2020) and targeting interventions to stroke early stages is the optimal preventive measure. Therefore, we focused our sequencing efforts on the early stages of stroke to identify therapeutic targets and characterize early cellular changes.

As microglial activation is traditionally recognized to play a deleterious role in ischemic stroke, inhibition of this step could alleviate ischemic brain injuries (Sofroniew, 2009; Wu, 2014). Currently, there is growing evidence that microglia differentiate into M1 and M2 types upon activation (Ma et al., 2017). Our results suggest that the M1–M2 dichotomy may be an oversimplified classification, representing only two extreme activation states. Subclusters 4, 9, and 10 are likely to be M1-type microglia, mainly enriched in the hypoxia pathway, and TNF-α-, IL- 6-, and IL-2-enriched inflammation-related genes and pathways. On the other hand, subclusters 6 and 8 exhibited a different response, with no significant M2-type marker genes (such as *Arg1*, *Ym1*, and *IL-10*) overexpressed. The above findings indicate that M2-type microglia cells may differentiate more slowly than M1-type microglia. In addition to the classical M1 and M2 marker genes, we have identified numerous novel subcluster-specific genes (such as *Gadd45b* in subclusters 4 and 9). *Gadd45b* has been reported to alleviate infarct volume *via* the TGF-β-smad3 pathway after ischemic stroke induction (Zhang et al., 2019). Furthermore, the GSVA results of subclusters 6 and 8 (likely to be M2-type) indicate that these subclusters are mainly enriched in the Kras signaling pathway, which was closely associated with the survival of cancer cells in previous studies (Saad et al., 2019). Through this pathway, we infer that these microglia cells may have higher chances of survival in ischemic stroke. Therefore, there is an urgent need in the acute stage of ischemic stroke to drive microglial polarization into a protective phenotype while inhibiting M1-type activation. In particular, M2-phenotypic microglia may be associated with restorative processes, neurogenesis, axonal remodeling, and angiogenesis (Hu et al., 2015). Compared with the sham group, microglia cells from the MCAO group were also enriched in phagocytosis processes. However, whether microglial phagocytosis plays a protective or damaging role in neurodegenerative diseases is still controversial (Fu et al., 2014). Microglial phagocytosis is a “double-edged sword”: on the one hand, it facilitates the repair of neurological damage; on the other, it induces or exacerbates neurodegenerative lesions in certain conditions (Hu et al., 2015).

In addition, peripheral macrophages are recruited to the lesions through the damaged BBB (Cowell et al., 2002). This process occurs within 24 h. Peripheral macrophage polarization seems to be complete, in contrast to microglia cells, indicating that brain resident microglia and blood immune cells have different activation periods after an ischemic stroke. Infiltration and polarization of macrophages can be detected in early stages of stroke, facilitating inflammation progression (Bernstein and Rom, 2020). Thus, interference with the initial peripheral immune cell recruitment may be an efficient measure to alleviate stroke inflammation; however, despite some improvement, the positive results from previous studies on this strategy were not significant (Vogelgesang et al., 2019). Moreover, previous research has shown that *Mmp9* is highly expressed in microglia, causing hydrolyzation and blood vessel damage, leading to BBB alterations (Rosenkranz et al., 2020). Another study suggested that physical disruption of the BBB can be induced by matrix metalloprotease through the digestion of BBB matrix proteins (Ji et al., 2017). In our analysis, *Mmp9* and *Mmp8* were overexpressed in macrophages in the MCAO group; this finding suggests that breakdown of the BBB may also be due to peripherally recruited macrophages in addition to microglia in the penumbra. BBB disruption seems to be a delayed secondary phase of stroke lesions. However, there is still controversy as to whether peripheral macrophages damage or protect the BBB.

Pathophysiological hallmarks of ischemic stroke are astrocyte activation and gliosis, which may help to restrain the ischemic core region during the early stages of an ischemic insult and positively affect the outcome after a stroke (Liu and Chopp, 2016). As part of the glial system, astrocytes and oligodendrocytes offer many essential biological functions, such as BBB formation, neuronal energy metabolism, structural maintenance, and intercellular communication (Jiang et al., 2018). Compared with microglia and macrophages, subclusters of these cells showed lesser polarization in the early stages of stroke in the scRNA-seq analysis. When astrocyte gap junctions are damaged during a stroke, inflammatory mediators such as cytokines and chemokines are released through the BBB outside the cell (Ma et al., 2018). In our results, both *Cyr61* in astrocytes and *Sgk3* in oligodendrocytes are overexpressed and these genes could be potential therapeutic targets in this phase. Despite the lack of research on ischemic stroke, previous studies have suggested that the *Cyr61* gene is closely associated with the proliferation of stressed and tumoral cells (Schwarz et al., 2002; Xie et al., 2019; Sun et al., 2020). Overexpression of the *Cyr61* gene may help astrocytes survive under acute ischemic stroke conditions. Additionally, GSVA showed oligodendrocyte subcluster 9 was enriched in IL-6, complement, TNF-α pathway, and Kras signaling, suggesting that *Sgk3* from oligodendrocytes may play an important role regulating oligodendrocyte viability and inflammatory responses during the acute stage of ischemic stroke (Inoue et al., 2016). Previous research showed that reactive astrocytes might be detrimental to neurological outcomes after stroke (Sofroniew, 2009). However, our scRNA-seq results demonstrate that some reactive astrocytes and oligodendrocytes in synergy exert beneficial effects.

In addition to astrocytes and oligodendrocytes, endothelial cells are also involved in the composition of the BBB (Rosenkranz et al., 2020). Results from scRNA-seq analysis showed that endothelial cells promote vascular reconstruction and extracellular communication after an ischemic injury. In addition, endothelial cells upregulated oxidative phosphorylation, ROS detoxification, glutathione metabolism, and cyclic nucleotide metabolism. The above findings suggest that endothelial cells may help the positive response to stroke, and may be an early therapeutic target.

All the cell subpopulations we studied in the ischemic penumbra promote neuron rescue and attempt to restore neuronal function. Our GO and KEGG analysis findings also revealed that neurons themselves are active in regeneration and nervous system development. On the other hand, other cellular subtypes with insufficient cells in our samples could not be analyzed, and their functions remain unclear.

## Conclusion

We identified several cell types and subtypes in the ischemic stroke penumbra, presenting an atlas of detailed information on a molecular and cellular level. Further research is needed to verify if targeting these molecules or cellular types could be developed into effective stroke treatments; other cells expressing identical targets could also be affected. However, further understanding of the activity of the proteins expressed by each cell type would facilitate the development of novel and effective therapeutic strategies to treat ischemic stroke. Meanwhile, research at the single-cell level will help us find more specific therapeutic targets and applications. Our single-cell study provides a new perspective on the pathological processes involved in ischemic stroke.

## Data Availability Statement

The datasets generated for this study can be found in online repositories. The names of the repository/repositories and accession number(s) can be found in the article/Supplementary Material.

## Ethics Statement

The animal study was reviewed and approved by Ethics Committee of the Fourth Military Medical University.

## Author Contributions

KG, ZiZ, and ZeZ carried out this project and designed the study. KG and JL performed MCAO surgery and Brain tissue dissociation. XWa and KG performed RNAscope *in situ* hybridization and immunofluorescence staining. WC, KT, and XWu carried out statistical analysis and interpreted the data. DF and JL helped in drafting the manuscript. LW, LX, BW, and YH revised the manuscript. All authors contributed to the article and approved the submitted version.

## Conflict of Interest

The authors declare that the research was conducted in the absence of any commercial or financial relationships that could be construed as a potential conflict of interest.

## Footnotes

1 http://biocc.hrbmu.edu.cn/CellMarker

## Abbreviations

DAPI: 4,6-diamidino-2-phenylindole
BBB: blood–brain barrier
FDR: false discovery rate
GO: gene ontology
GSVA: gene set variation analysis
KEGG: Kyoto Encyclopedia of Genes and Genomes
MCAO: middle cerebral artery occlusion
NVU: neurovascular unit
OPC: oligodendrocyte progenitor cells
PBS: phosphate-buffered saline
PCA: principal component analysis
scRNA-seq: single-cell RNA sequencing
SCENIC: single-cell regulatory network inference and clustering
t-SNE: t-distributed stochastic neighbor embedding
TPM: transcript per million
UMI: unique molecular identifier.
