# Supplementary figures and images for: Single-Cell RNA Sequencing With Combined Use of Bulk RNA Sequencing to Reveal Cell Heterogeneity and Molecular Changes at Acute Stage of Ischemic Stroke in Mouse Cortex Penumbra Area
Source: Front Cell Dev Biol. 2021 Feb 22;9:624711. doi: 10.3389/fcell.2021.624711 (PMC7937629; doi:10.3389/fcell.2021.624711)

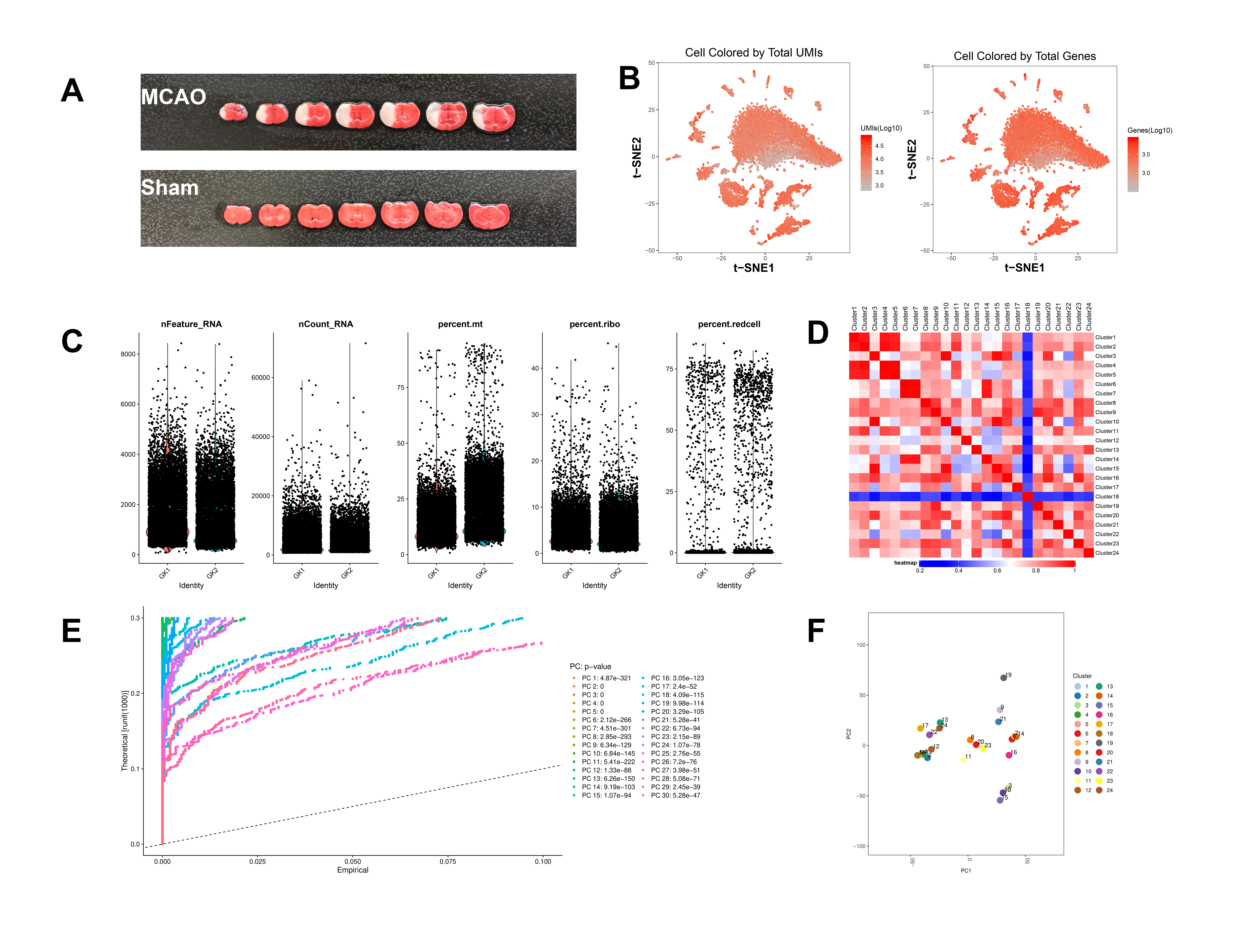

Supplement: Supplementary file 1 [file Image_1.TIF]

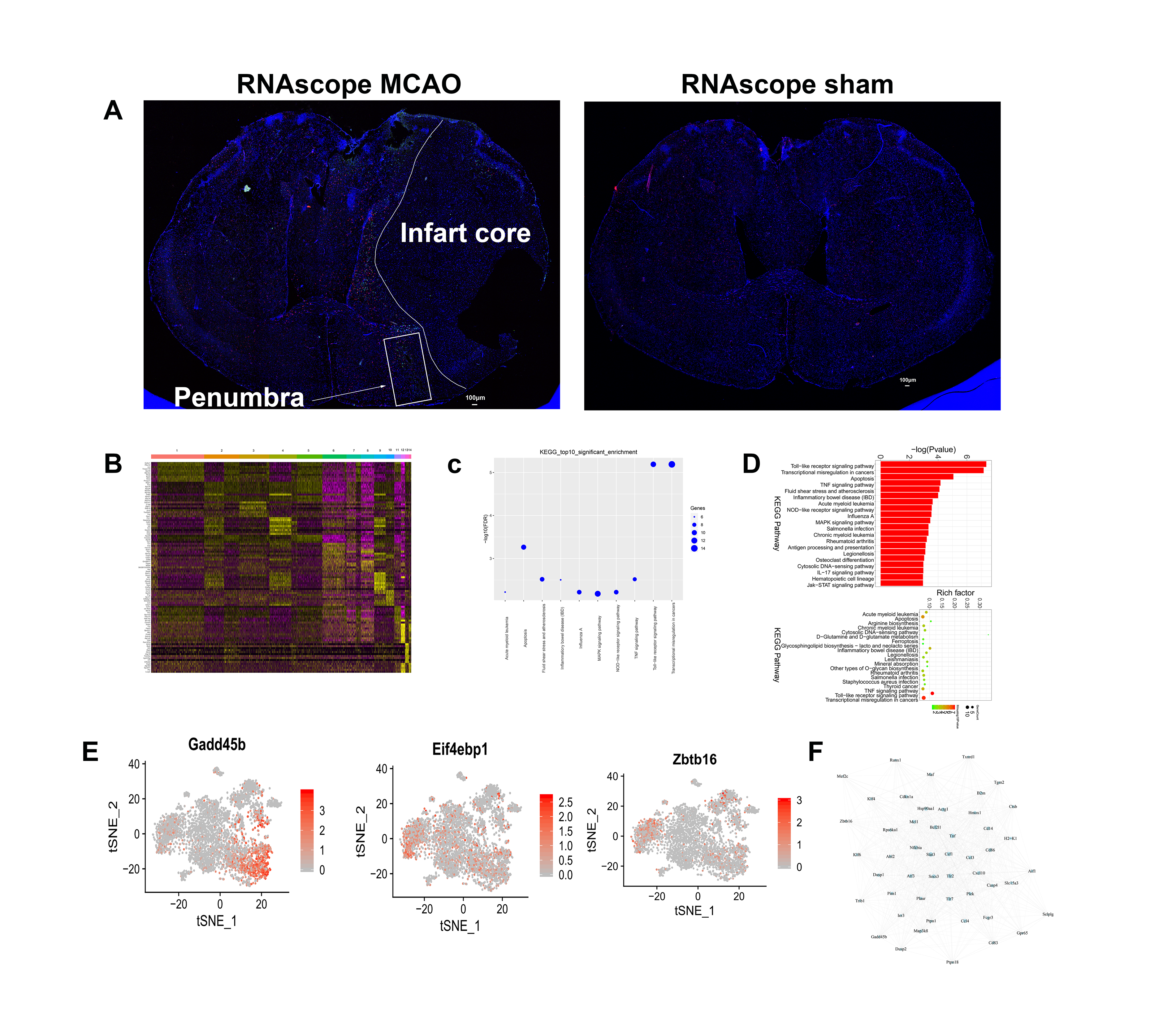

Supplement: Supplementary file 2 [file Image_2.TIF]

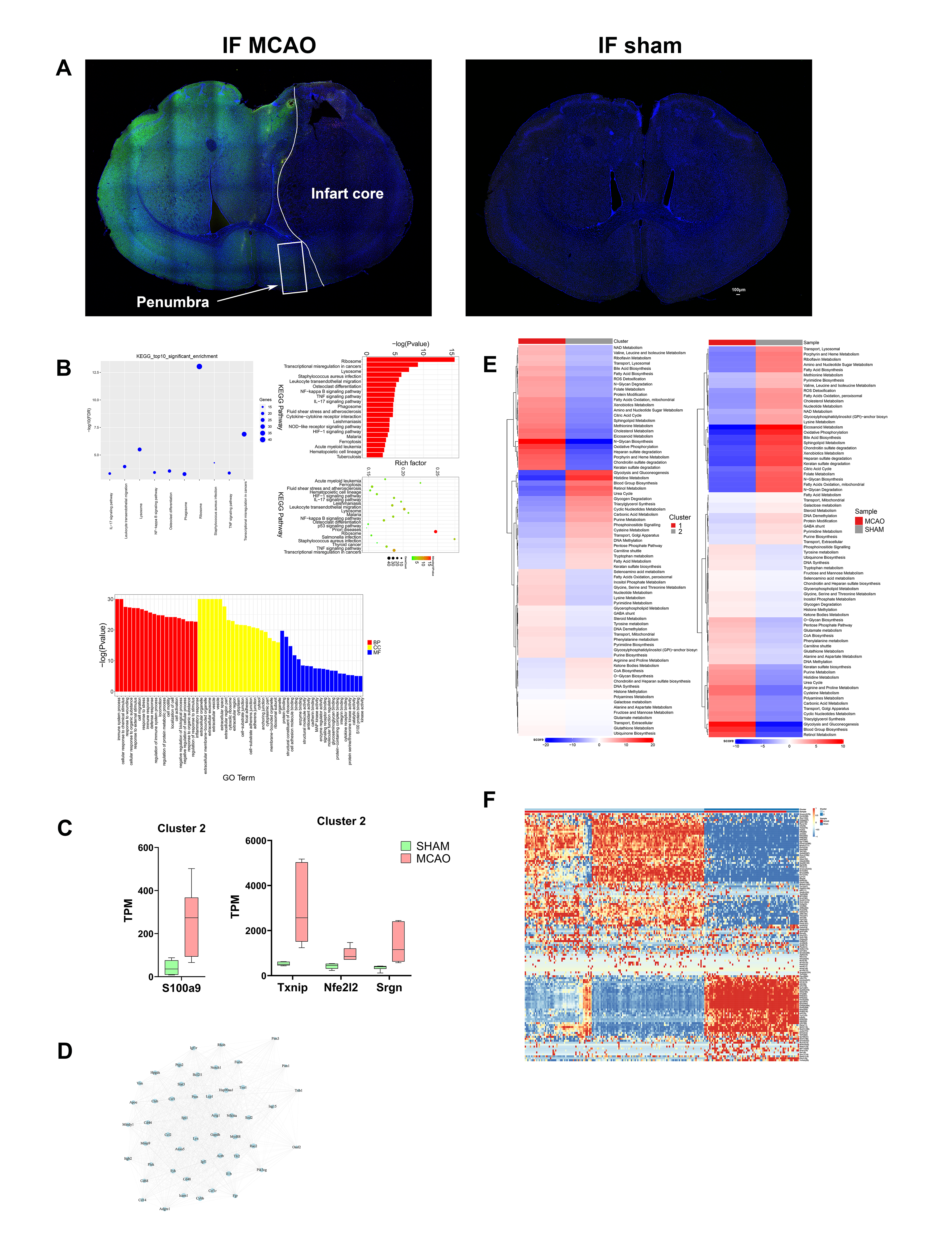

Supplement: Supplementary file 3 [file Image_3.TIF]

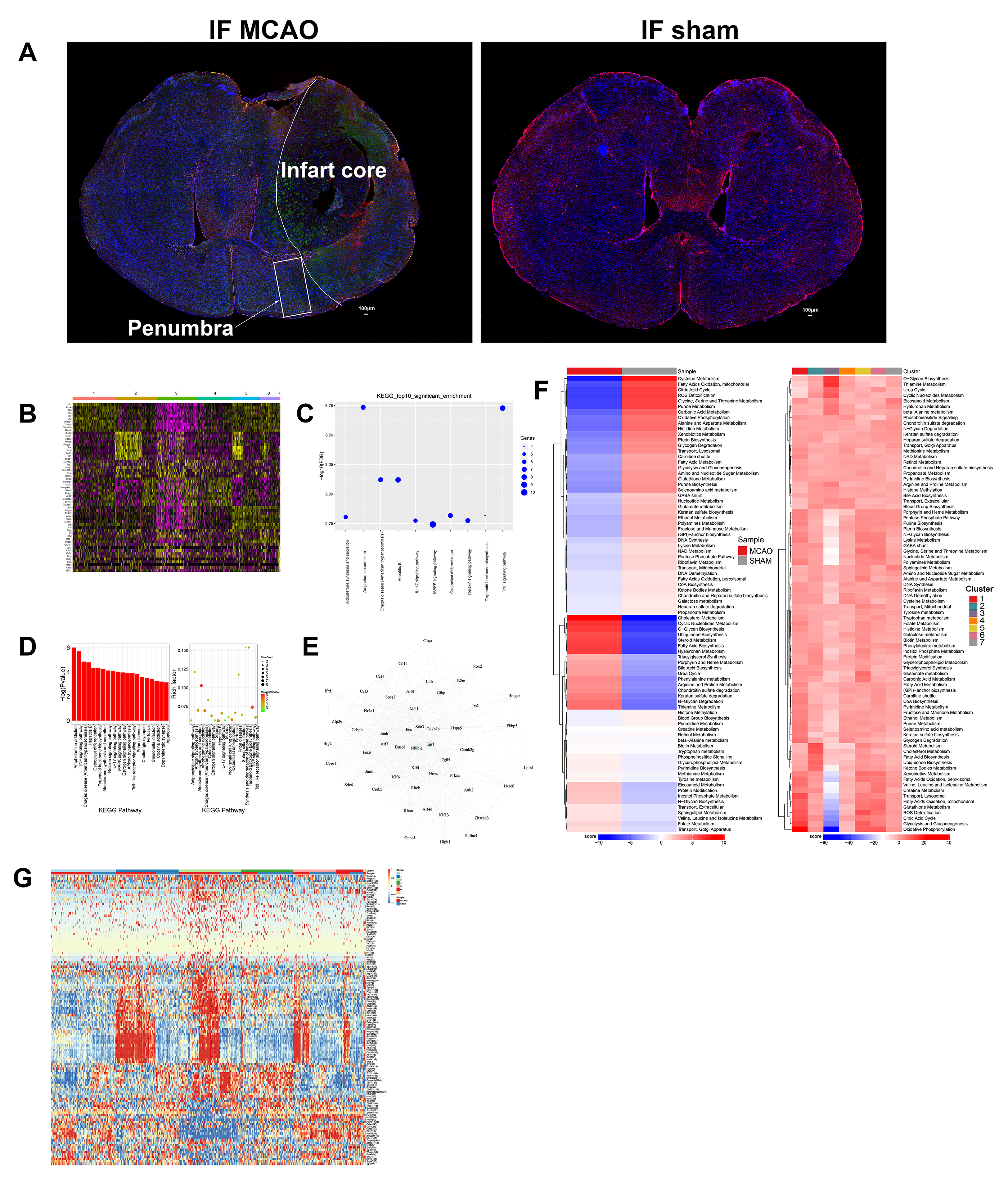

Supplement: Supplementary file 4 [file Image_4.TIF]

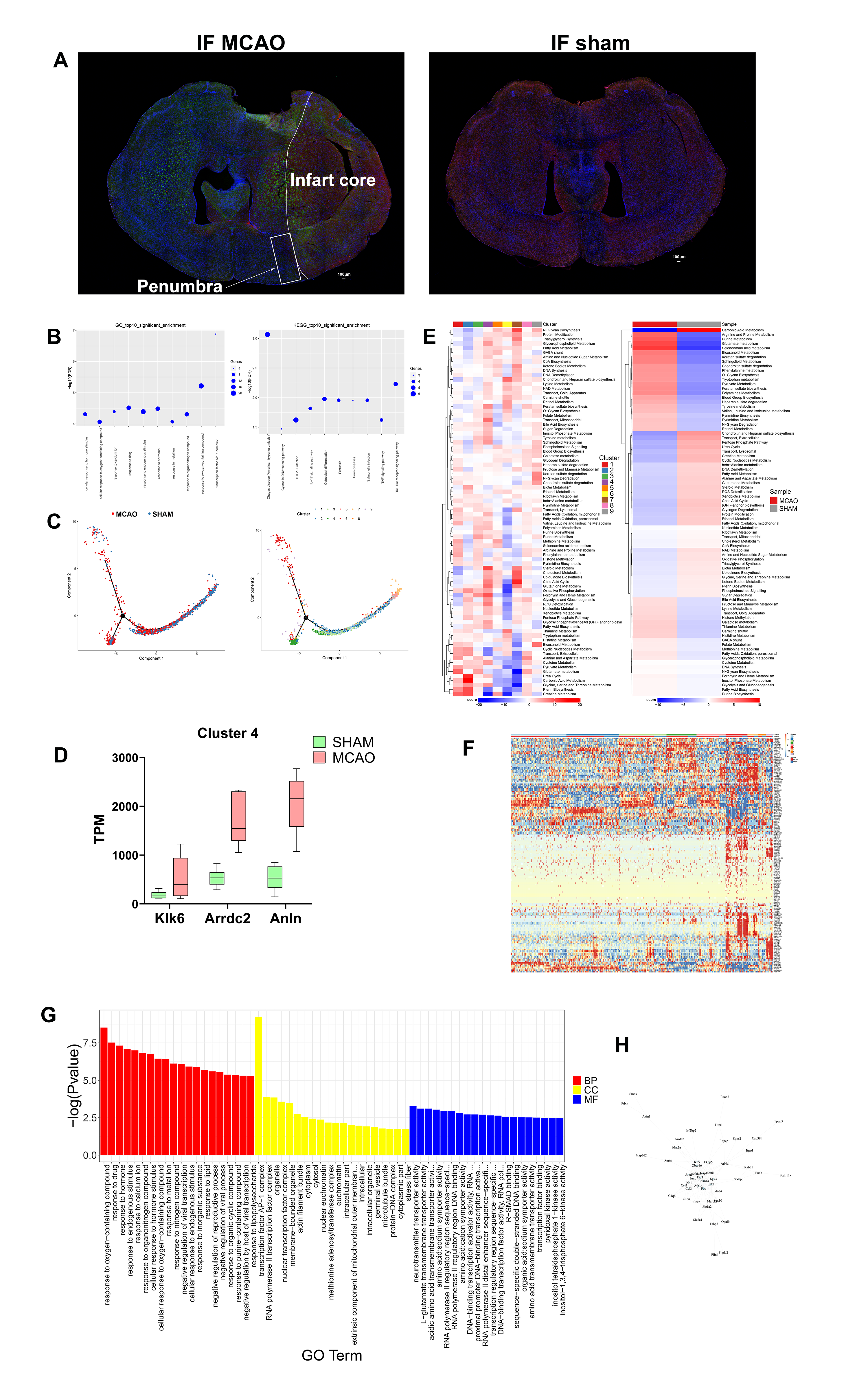

Supplement: Supplementary file 5 [file Image_5.TIF]

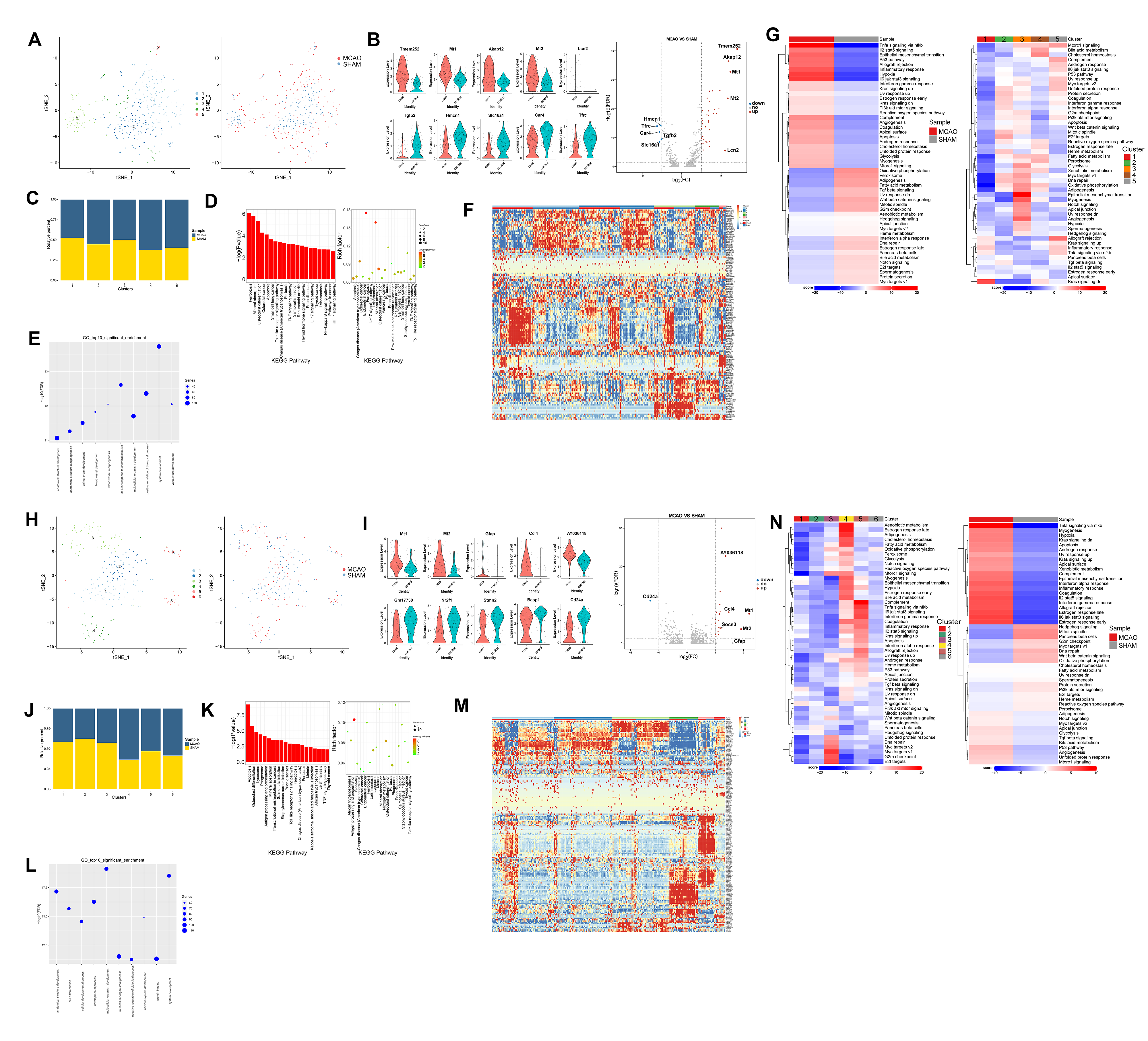

Supplement: Supplementary file 6 [file Image_6.TIF]

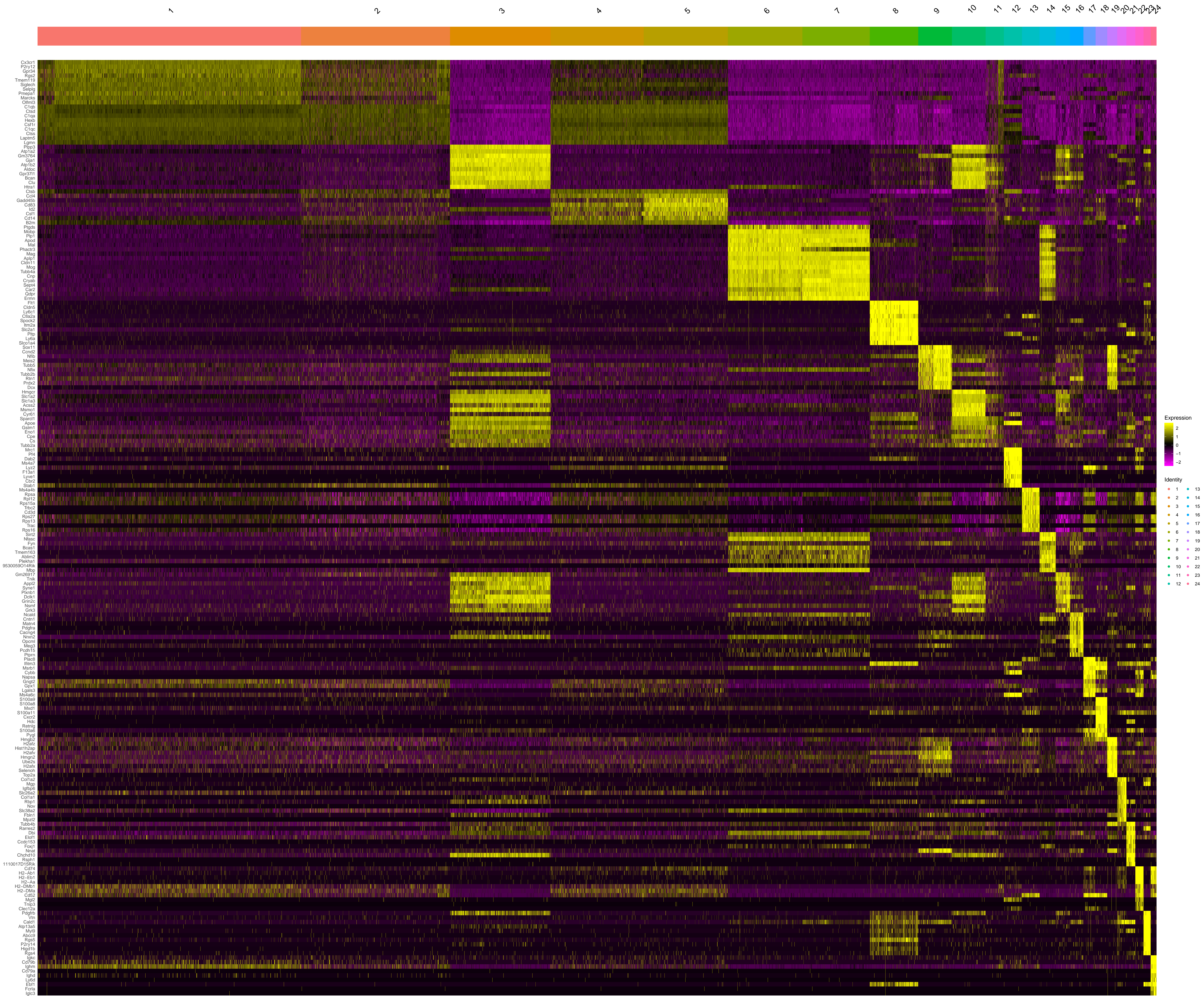

Supplement: Supplementary file 7 [file Presentation_1.PDF]

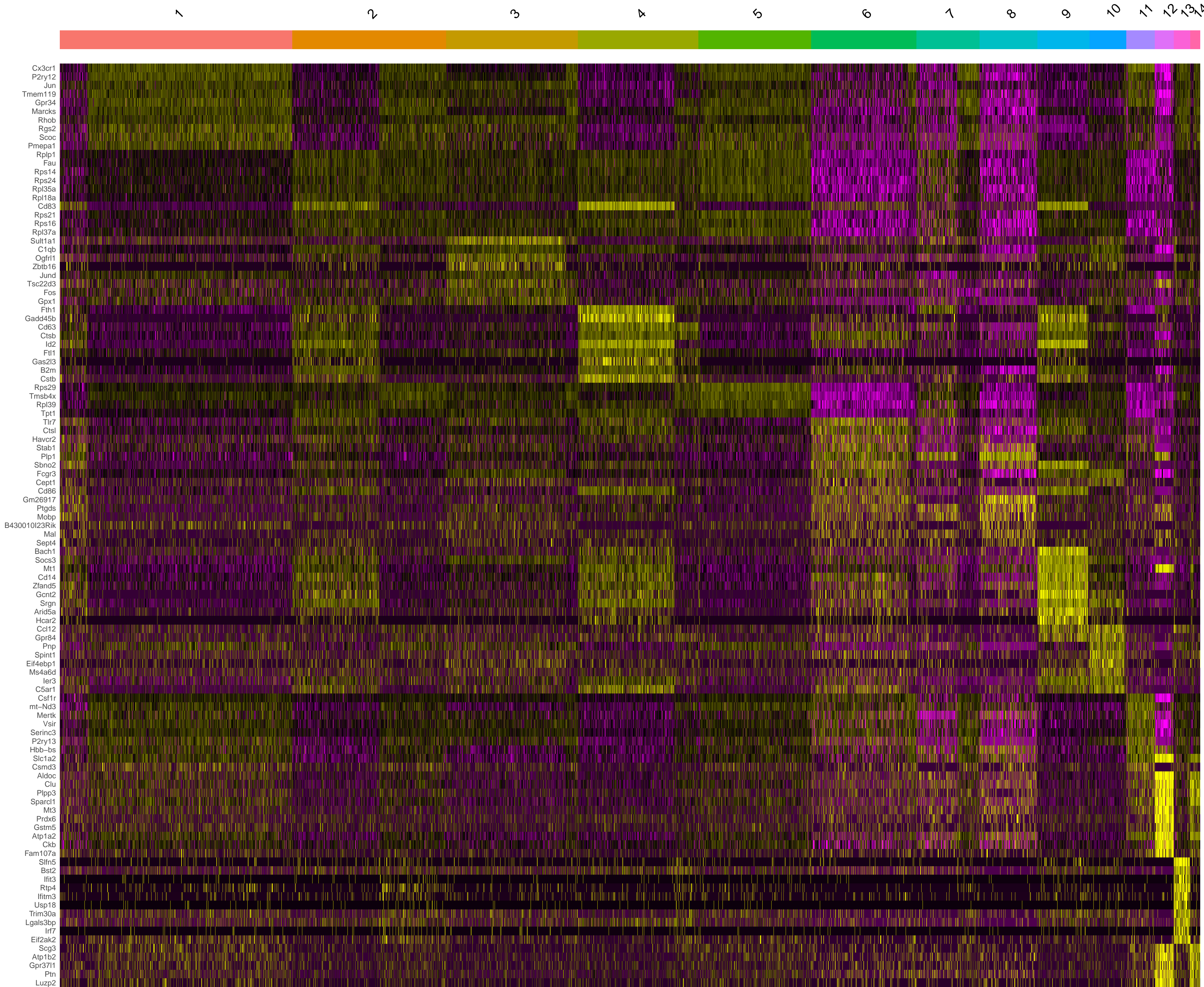

Supplement: Supplementary file 8 [file Presentation_2.PDF]

1

2

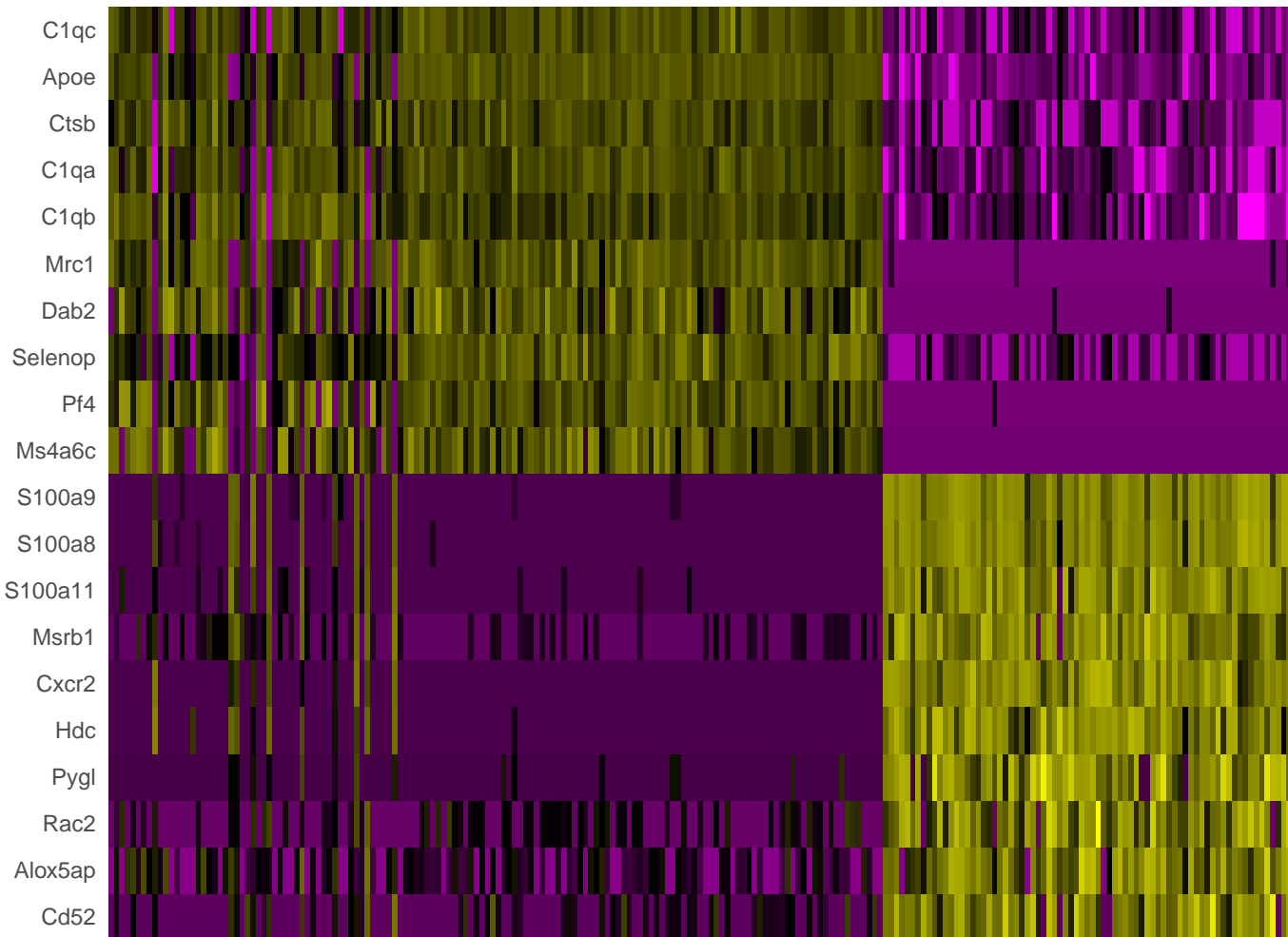

Supplement: Supplementary file 9 [file Presentation_3.PDF]

1

2

3

4

5

6

7

8

9

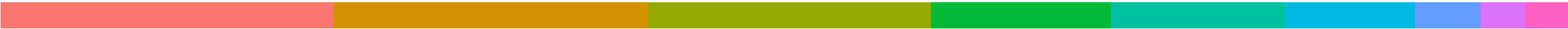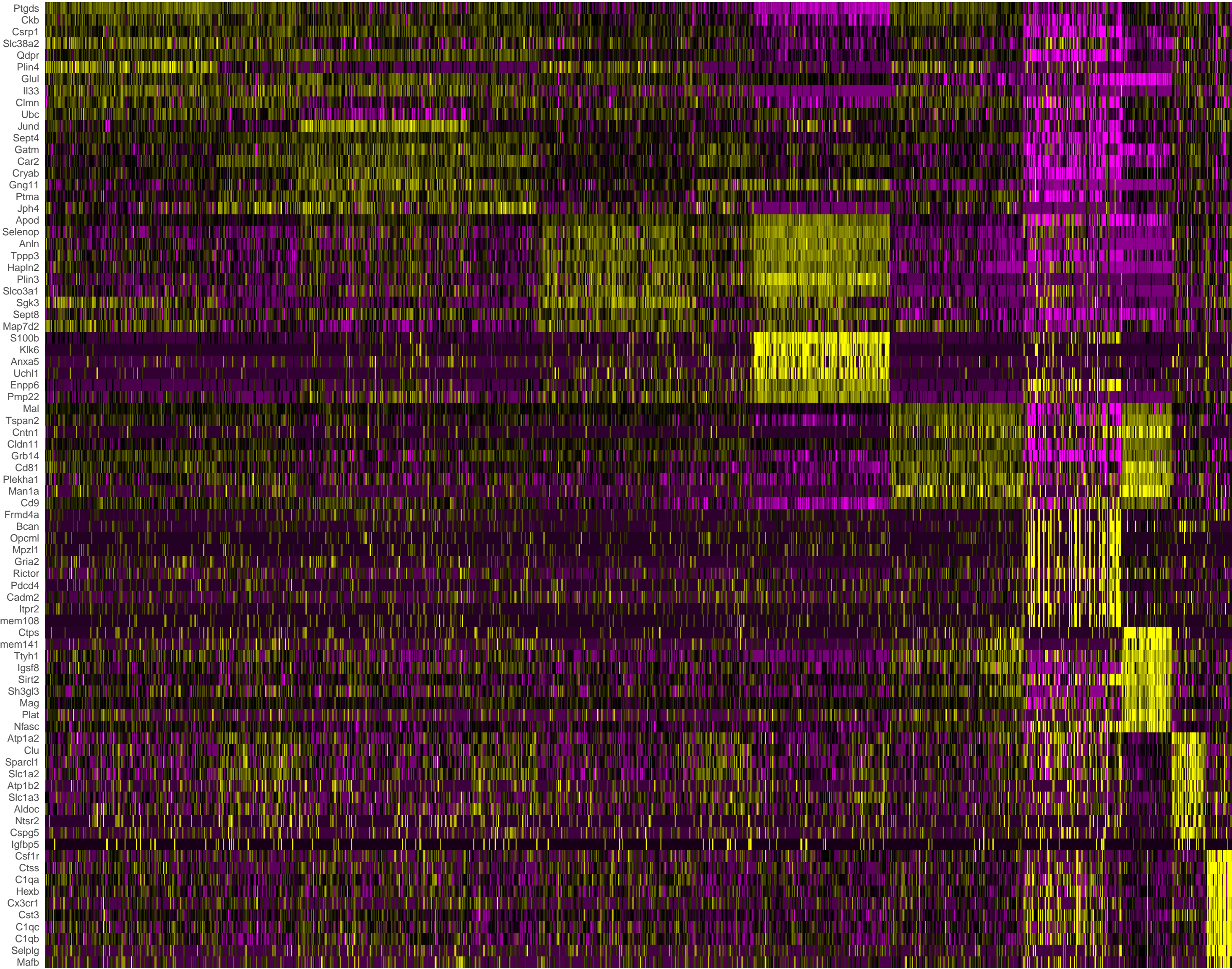

Supplement: Supplementary file 11 [file Presentation_5.PDF]

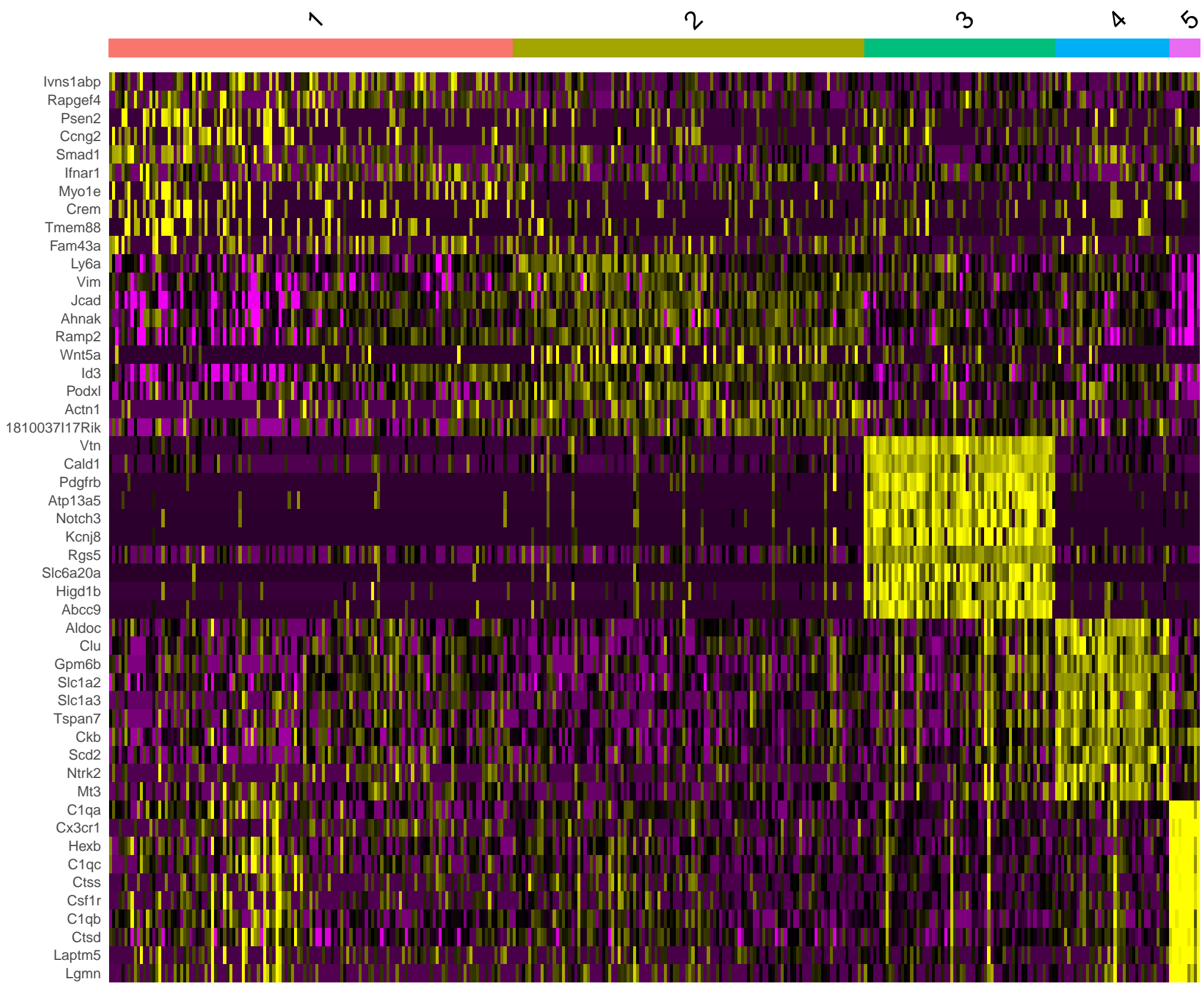

Supplement: Supplementary file 12 [file Presentation_6.PDF]

1

2

3

4

5

6

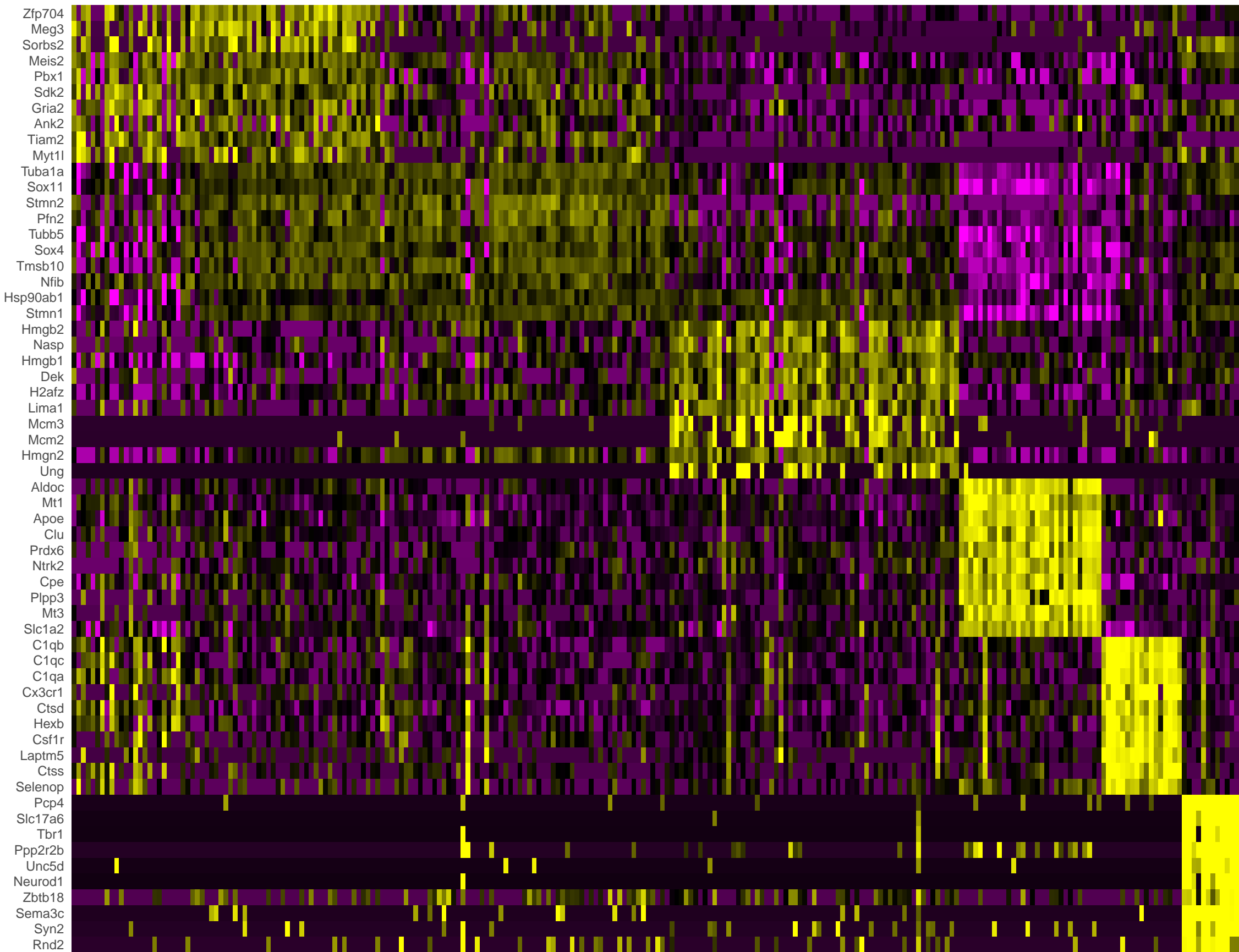

Supplement: Supplementary file 13 [file Presentation_7.PDF]
