# Supplementary material for: Single-Cell RNA Sequencing With Combined Use of Bulk RNA Sequencing to Reveal Cell Heterogeneity and Molecular Changes at Acute Stage of Ischemic Stroke in Mouse Cortex Penumbra Area
Source: Front Cell Dev Biol. 2021 Feb 22;9:624711. doi: 10.3389/fcell.2021.624711 (PMC7937629; doi:10.3389/fcell.2021.624711)
Supplement: Supplementary file 10 [file Presentation_4.PDF]

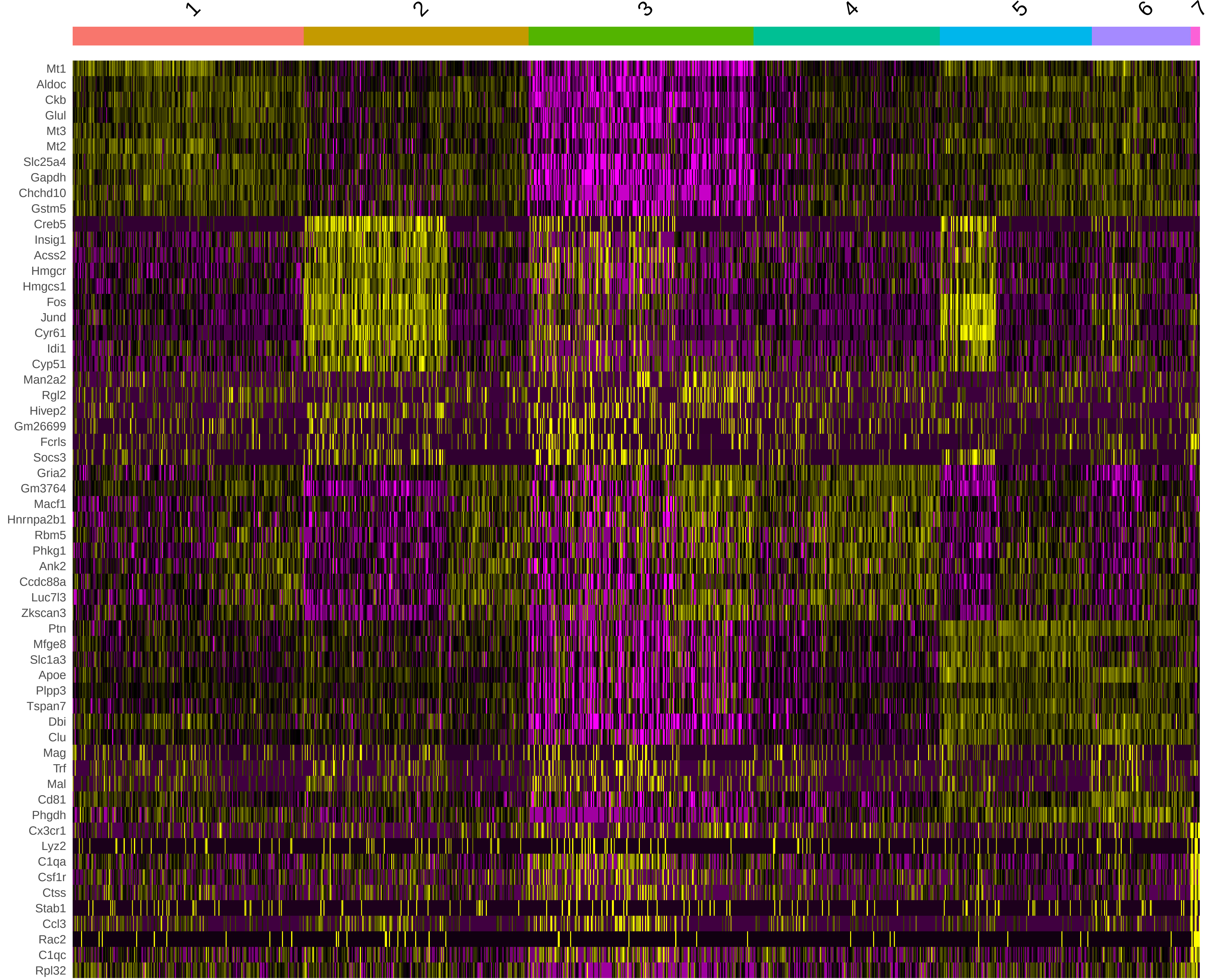
1
2
3
4
5
6
7
Mt1
Aldoc
Ckb
Glul
Mt3
Mt2
Slc25a4
Gapdh
Chchd10
Gstm5
Creb5
Insig1
Acss2
Hmgcr
Hmgcs1
Fos
Jund
Cyr61
Idi1
Cyp51
Man2a2
Rgl2
Hivep2
Gm26699
Fcrls
Socs3
Gria2
Gm3764
Macf1
Hnrnpa2b1
Rbm5
Phkg1
Ank2
Ccdc88a
Luc7l3
Zkscan3
Ptn
Mfge8
Slc1a3
Apoe
Plpp3
Tspan7
Dbi
Clu
Mag
Trf
Mal
Cd81
Phgdh
Cx3cr1
Lyz2
C1qa
Csf1r
Ctss
Stab1
Ccl3
Rac2
C1qc
Rpl32
