# Supplementary material for: Single-Cell RNA Sequencing With Combined Use of Bulk RNA Sequencing to Reveal Cell Heterogeneity and Molecular Changes at Acute Stage of Ischemic Stroke in Mouse Cortex Penumbra Area
Source: Front Cell Dev Biol. 2021 Feb 22;9:624711. doi: 10.3389/fcell.2021.624711 (PMC7937629; doi:10.3389/fcell.2021.624711)
Supplement: Supplementary file 14 [file Table_1.DOCX]

**Supplementary Fig. 1. (A)** Mouse brain slices from MCAO and Sham groups. **(B)** Total UMIs and genes per cell shown in t-SNE plot. **(C)** Number of genes, number of UMI sequences, and proportion of UMI sequences from mitochondrial and ribosomal genes (from left to right) per cell between MCAO and sham group. **(D)** Correlation analysis of 23 subclusters. **(E)** JackStrawPlot shows the p-Value of each principle component. **(F)** PCA based on cell clusters revealed by cell marker genes.

**Supplementary** **Fig. 2. (A)** Whole brain scan of RNAscope in situ hybridization in the MCAO and sham mouse. The square area (sampling zone of sequencing) in the picture is the zoomed area of Fig. 2h. **(B)** Heatmap of top10 differential expression genes in each cell subcluster of microglia cells. **(C, D, E)** KEGG enrichment analysis of microglia cells differential expression genes. **(F)** String diagram of differential expression genes of microglia cells between MCAO and sham group.

**Supplementary** **Fig. 3. (A)** Whole brain scan of RNAscope in situ hybridization in the MCAO and sham mouse. The square area (sampling zone of sequencing) in the picture is the zoomed area of Fig. 3h. **(B)** GO and KEGG enrichment analysis of peripheral macrophage cells differential expression genes. **(C)** Validation of scRNA-seq results for differential expression genes in subcluster 2 of peripheral macrophage cells in bulk RNA-seq. **(D)** String diagram of differential expression genes of peripheral macrophages between MCAO and sham group. **(E)** Analysis of metabolic pathways between MCAO and sham groups and each subclusters. **(F)** SCENIC analysis displayed the variance of transcription factors between each subcluster of peripheral macrophage cells.

**Supplementary Fig. 4.** **(A)** Whole brain scan of RNAscope in situ hybridization in the MCAO and sham mouse. The square area (sampling zone of sequencing) in the picture is the zoomed area of Fig. 4h. **(B)** Heatmap of top10 differential expression genes in each cell subcluster of astrocyte cells. **(C, D)** KEGG enrichment analysis of astrocyte cells differential expression genes. **(E)** String diagram of differential expression genes of astrocyte cells between MCAO and sham group. **(F)** Analysis of metabolic pathways between MCAO and sham groups and each subclusters. **(G)** SCENIC analysis displayed the variance of transcription factors between each subcluster of astrocyte cells.

**Supplementary** **Fig. 5. (A)** Whole brain scan of RNAscope in situ hybridization in the MCAO and sham mouse. The square area (sampling zone of sequencing) in the picture is the zoomed area of Fig. 5g. **(B, G)** Dot plot of GO and KEGG enrichment analysis of Oligodendrocyte cells differential expression genes. **(C)** Differentiation trajectories of subclusters of oligodendrocyte cells in ischemic stroke. **(D)** Validation of scRNA-seq results for differential expression genes (Klk6, Arrdc2 and Anln) in subcluster 4 of oligodendrocyte cells in bulk RNA-seq. **(E)** Analysis of metabolic pathways between MCAO and sham groups and each subclusters. **(F)** SCENIC analysis displayed the variance of transcription factors between each subcluster of oligodendrocyte cells. **(H)** String diagram of differential expression genes of oligodendrocyte cells between MCAO and sham group.

**Supplementary Fig. 6.** **Endothelial cells and Neurons in MCAO and sham group. (A)** t-SNE map of 354 endothelial cells with the associated cell subcluster (left) and its sample type of origin (right). **(B)** Violin and volcano plot showing the top 10 differential expression genes of endothelial cells between MCAO and sham groups. **(C)** Proportion of each cell subclusters of endothelial cells in MCAO and sham groups. **(D, E)** GO and KEGG enrichment analysis of oligodendrocyte cells differential expression genes. **(F)** SCENIC analysis displayed the variance of transcription factors between each subcluster of endothelial cells. **(G)** Differences in pathway activities scored per cell by GSVA between MCAO and normal endothelial cells and between each subcluster. **(H)** t-SNE map of 246 neuron cells with the associated cell subcluster (left) and its sample type of origin (MCAO or sham group). **(I)** Violin and volcano plot showing the top 10 differential expression genes of neuron cells between MCAO and sham groups. **(J)** Proportion of each cell subclusters of endothelial cells in MCAO and sham groups. **(K, L)** GO and KEGG enrichment analysis of neuron cells differential expression genes. **(M)** SCENIC analysis displayed the variance of transcription factors between each subcluster of endothelial cells. **(N)** Differences in pathway activities scored per cell by GSVA between MCAO and normal neuron cells and between each subcluster.
